# Effect of Lipidation on the Structure, Oligomerization, and Aggregation of Glucagon-like Peptide 1

**DOI:** 10.1021/acs.bioconjchem.4c00484

**Published:** 2025-01-22

**Authors:** Eva Přáda Brichtová, Irina A. Edu, Xinyang Li, Frederik Becher, Ana L. Gomes dos Santos, Sophie E. Jackson

**Affiliations:** † Yusuf Hamied Department of Chemistry, University of Cambridge, Cambridge CB2 1EW, U.K.; ‡ Now: Institute of Chemical, Environmental and Bioscience Engineering, Technische Universität Wien, Gumpendorferstraße 1A, Vienna 1060, Austria; § Advanced Drug Delivery, Pharmaceutical Sciences, R&D, AstraZeneca, Biomedical Campus, Cambridge CB2 0AA, U.K.

## Abstract

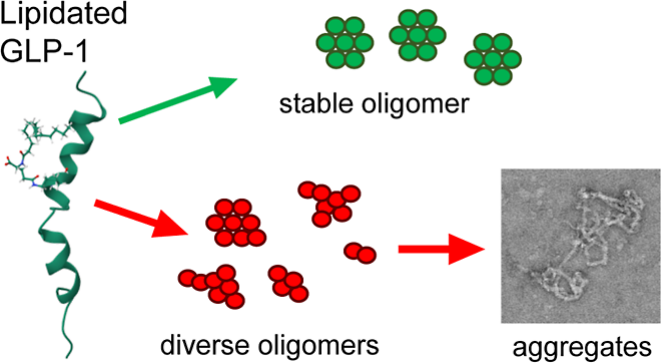

Lipidated analogues of glucagon-like peptide 1 (GLP-1) have gained enormous attention as long-acting peptide therapeutics for type 2 diabetes and also antiobesity treatment. Commercially available therapeutic lipidated GLP-1 analogues, semaglutide and liraglutide, have the great advantage of prolonged half-lives *in vivo* of hours and days instead of minutes as is the case for native GLP-1. A crucial factor in the development of novel lipidated therapeutic peptides is their physical stability, which greatly influences manufacturing and drug product development. This work provides a systematic study of the solubility, structure, oligomerization, and long-term stability of five different lipidated analogues of GLP-1, varying in the position of the lipidation site and the nature of lipid attachment. The lipidation was found to negatively impact the peptide solubility, in all cases, limiting it to a specific pH range. An increase in the α-helical secondary structure was observed upon lipidation, and the lipidated analogues were found to form larger and more stable oligomeric species compared to nonlipidated GLP-1. Importantly, the distributions and populations of oligomeric species formed were regulated by both the position and the nature of the lipidation. During the 6 days of sample aging, several lipidated analogues formed aggregates with variable morphologies ranging from elongated mature fibrils to amorphous structures. The kinetics of aggregation often showed multiple steps and did not follow a standard nucleation-propagation mechanism. A wide range of behaviors was observed, and while our observations indicate that the formation of a single stable oligomer results in the greatest physical stability, positioning the lipid group toward the N-terminus of the peptide results in extremely rapid amyloid formation. We believe that our study provides important findings for the development of long-acting lipidated analogues of peptide therapeutics.

## Introduction

The usage of many peptide-based biopharmaceuticals is limited by their short half-lives *in vivo*.^1,2^ Several types of modifications of peptide-based biopharmaceuticals have been developed to increase stability and proteolytic resistance of these molecules and extend their half-lives.^3^ Peptide lipidation has been proven to be an effective strategy with several marketed peptide-based drugs such as liraglutide,^4^ semaglutide,^5^ tirzepatide,^6^ long-acting insulin detemir,^7^ or somapacitan.^8^ Mechanisms underlying the half-life extension of lipidated therapeutic peptides *in vivo* include an increased tendency to self-assemble into larger oligomers as well as binding to human serum albumin. These mechanisms reduce the enzymatic degradation of the peptides and slow their renal clearance.^1,3,9−13^

Glucagon-like peptide-1 (GLP-1) is a peptide hormone stimulating a decrease in blood glucose levels and thus is of crucial importance as a therapeutic agent for the treatment of type 2 diabetes and obesity. However, native GLP-1 has a half-life *in vivo* of circa 2 min, which results from rapid degradation by the dipeptidyl peptidase-4 enzyme.^14,15^ It was shown that lipidation of GLP-1 can increase its half-life *in vivo* to hours or even days. Nowadays, two lipidated analogues of GLP-1 are available on the market-liraglutide (Saxenda or Victoza) and semaglutide (Ozempic/Rybelsus or Wegovy).^3−5,16^ Liraglutide has a palmitic acid (C16) linked to Lys20 via a γ-glutamic acid spacer and a substitution of Arg → Lys at position 28. Pharmacokinetic data showed that liraglutide has a prolonged half-life of about 9–13 h which allows once-daily injections of the therapeutic.^4^ Semaglutide has an amino acid substitution Ala2 → Aib2 in addition to Arg28 → Lys28 substitution, a stearic diacid (C18) lipidation at Lys20 and a longer optimized spacer connecting the lipid with the Lys20 side chain. These modifications in semaglutide result in an *in vivo* half-life of 7 days, allowing for once-weekly administration of the drug. Semaglutide is available for both oral and subcutaneous administration.^3,5^

Although these lipidated GLP-1 analogues have been widely characterized in terms of their pharmacokinetic properties, there have been only a few studies characterizing their biophysical properties and physical stability.^17−21^ Information on, and understanding of, the factors governing physical stability are essential for optimizing drug manufacturing, development, and storage. Our study aims to systematically rationalize the effect of lipidation on the biophysical properties of GLP-1 and its stability and aggregation behavior. In this work, the term “self-assembly” refers to the formation of higher molecular weight species, which is not accompanied by the change in the secondary structure of the peptide. In contrast, the term “aggregation” in this work is used for a self-assembly process, which includes changes in the secondary structure of the peptide. The self-assembly processes occur shortly after dissolution of the lyophilized powder of peptide analogue, while the aggregation events are mainly observed after prolonged incubation.

For many systems, self-assembly is peptide concentration-dependent, and these amphiphilic molecules self-assemble above a certain concentration threshold, which is known as the critical aggregation concentration (cac).^11^ Conjugation of a hydrophobic lipid chain to a peptide has been observed to often lead to the stabilization or even induction of the secondary structure.^22,23^ This is due to the fact that these amphiphilic molecules tend to self-assemble/oligomerize rapidly, which increases the local concentration of the peptide resulting in an increase in intramolecular noncovalent interactions which can help stabilize specific secondary structures.^24,25^ This effect has mostly been demonstrated for α-helical peptides, e.g., for collagen-based peptides, and it was established that a series of dialkyl chains enhances the thermal stability of the α-helical conformation, compared to nonlipidated peptides.^26^

Upon aggregation, which is frequently associated with changes in the secondary structure of a peptide or protein, the peptide or protein usually loses its biological activity. Aggregates can have a highly regular structure, e.g., amyloid fibrils, or be amorphous in nature. In most cases, the presence of aggregates in biopharmaceutical formulations is nondesirable as it not only lowers the amount of active drug in the formulation but may also present a cytotoxicity/immunogenicity risk.^27,28^ On the other hand, the nanostructures formed by the aggregation of peptides and proteins are of a great interest in the field of biomaterial development^29,30^ and as long-acting, slow release drug formulations.^31,32^ Lipidated peptides and lipoproteins have been observed to aggregate and self-assemble into various morphologies such as micelles, bilayers, amyloid fibrils, nanotubes, or vesicles.^33−35^ It was previously shown that alkylation of amylin, a peptide that is prone to amyloid formation, can selectively regulate the morphology of observed aggregates or suppress their formation based on the length of the alkyl chain.^36^ A similar effect was observed for lipidated exendin-4-derived dual peptide agonists.^37^ Another study showed that self-assembly of toll-like receptor agonist lipopeptides into either spherical micelles or flexible worm-like micelles is dependent on the number of lipid chains attached to the peptide.^38^ Several studies also showed that the morphology of self-assembled species/aggregates can be regulated by pH, ionic strength, or temperature.^39−42^

Using a range of biophysical techniques, the effect of lipidation on the biophysical properties of GLP-1 analogues was investigated for five lipidated analogues differing in the position of lipidation or the nature of the lipid moiety. Specifically, the effect of lipidation on peptide solubility, secondary structure, self-assembly behavior, and long-term stability (aggregation behavior) was assessed. In the first part of the following study, the solubility of the lipidated analogues is tested, and the structure and oligomerization (self-assembly) behavior are assessed for the freshly prepared samples in biological buffers. The second part of the study deals with the long-term stability of the analogues and describes the kinetics of aggregation observed for all variants over longer periods of times (days). In addition, the morphology and structure of aggregates formed are studied by using spectroscopic techniques and electron microscopy.

## Results and Discussion

**Figure 1:**
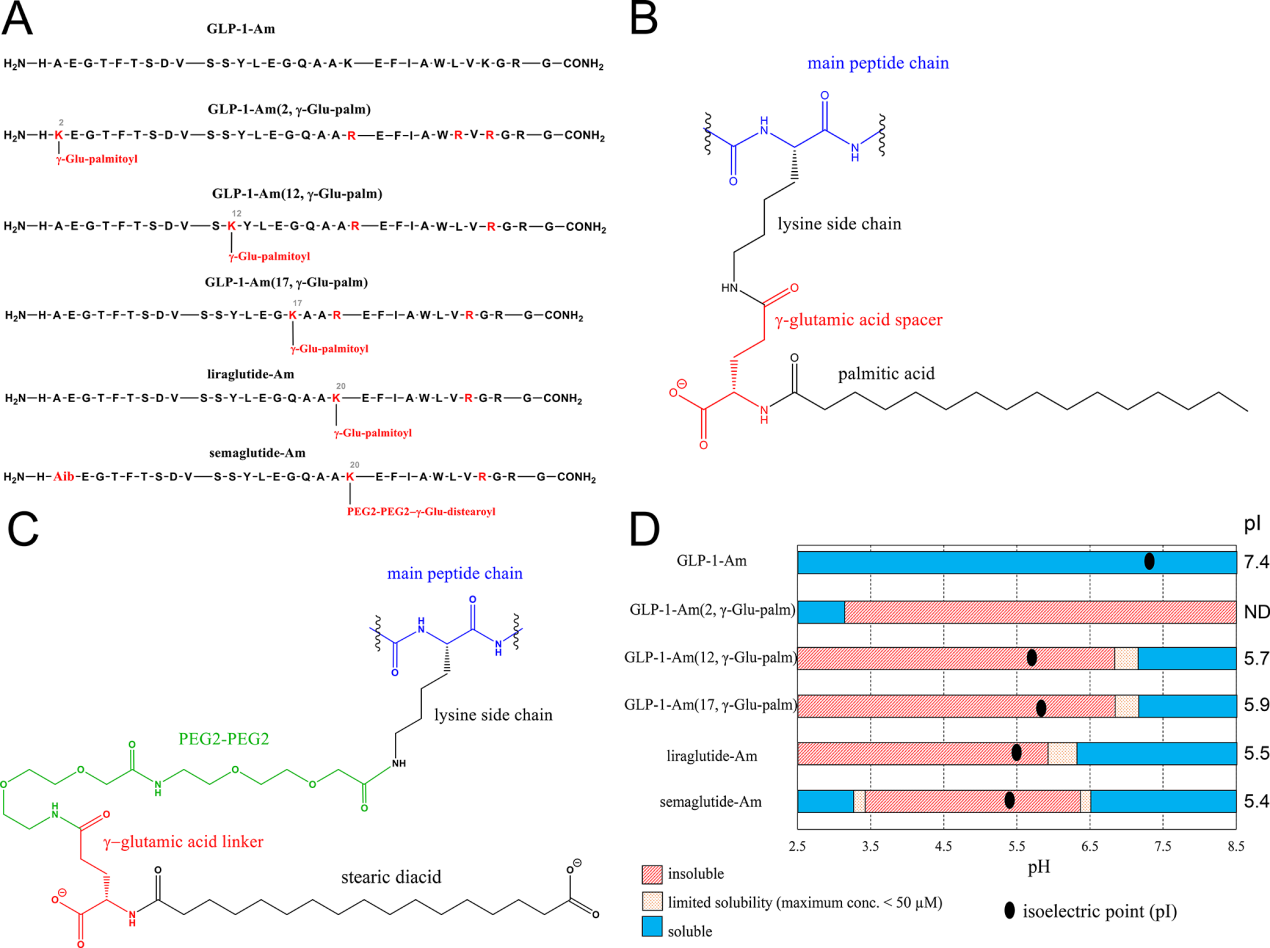
Structure and solubility of lipidated GLP-1-Am analogues. Sequence of GLP-1-Am and its lipidated analogues with the position of lipidation highlighted. Apart from the C-terminal amidation, liraglutide-Am and semaglutide-Am are identical to commercially available GLP-1 analogues liraglutide and semaglutide (A). Structural detail of the palmitic acid lipid moiety, linker, and attachment site (B). Structural detail of the stearic diacid lipid moiety, linker, and attachment site (C). pH-dependent solubility of lipidated GLP-1-Am analogues (D). The peptide under specific conditions is defined as soluble if concentrations above 50 μM can be achieved. The peptide is defined as insoluble if it is not possible to attain concentrations above 1 μM. Limited solubility corresponds to maximum concentrations ranging from 1 to 50 μM. The isoelectric points (pI) of the analogues are given next to the solubility chart and also marked by a black dot. The pI of GLP-1-Am (2, γ-Glu-palm) was not determined (ND) due to its low solubility.

In this work, five lipidated variants of C-terminally amidated glucagon-like peptide 1 (GLP-1-Am) were studied to establish the effect of the site of lipidation and the lipid moiety on the properties and physical stability of the peptide (Figure 1A). Four analogues contain a palmitic acid moiety attached via a γ-glutamic acid linker to a lysine side chain (Figure 1B) and vary in the position of lipidation. In addition, a C-terminally amidated variant of semaglutide, semaglutide-Am, containing a stearic diacid moiety attached via γ-glutamic acid and PEG2-PEG2 linker to a lysine side chain (Figure 1C) was also studied.

### Lipidation Limits the Solubility of GLP-1-Am Variants

The solubility of nonlipidated GLP-1-Am and its lipidated analogues was tested over a pH range from 2.5 to 8.5. Figure 1D illustrates the solubility of each lipidated analogue and nonlipidated GLP-1-Am at different pH values. While nonlipidated GLP-1-Am is soluble at all pH values tested, the solubility of lipidated analogues is pH-limited. The greatest solubility restriction was observed for GLP-1-Am (2, γ-Glu-palm), which is soluble only at around pH 3 and lower. GLP-1-Am (12, γ-Glu-palm), GLP-1-Am (17, γ-Glu-palm), and liraglutide-Am [GLP-1-Am (20, γ-Glu-palm), in previous notation] are soluble only at neutral and basic pH values with liraglutide-Am having the widest pH range of solubility: pH 6 to pH 8.5. Interestingly, semaglutide-Am [GLP-1-Am (20, PEG2-PEG2-γ-Glu-stear), in previous notation], shows two solubility windows, at around pH 3 and above pH 6.5. Isoelectric points (pI) of the analogues were determined using isoelectric focusing gel electrophoresis (Figure S1), and the determined values are given in Figure 1D in the column next to the solubility chart. The experimentally determined pI values were compared to the values which were theoretically calculated using individual p*K*_a_ and p*K*_b_ values of the ionizable groups—Figure S2 and Table S1, with the greatest deviation from the calculated values observed for liraglutide-Am and semaglutide-Am. It is interesting to note that these both form well-defined oligomeric states (see later sections), in which it is likely that the local chemical environment of the individual ionizable groups has changed. With the exception of the low pH solubility window for semaglutide-Am, the lipidated analogues were soluble only when their net charge was negative, i.e., at a pH above their pI values. Interestingly, not only the nature of the lipid moiety but also the position of the lipidation site was observed to affect the solubility of the analogues. Although there have been attempts to rationalize the solubility and even develop solubility predictors for peptide/protein analogs containing non-natural amino acids and amino acid derivatives, the prediction of the solubility of lipidated analogues remains challenging due to its complexity.^43,44^

### Lipidation Increases α-Helicity of GLP-1-Am and Promotes Oligomerization

**Figure 2:**
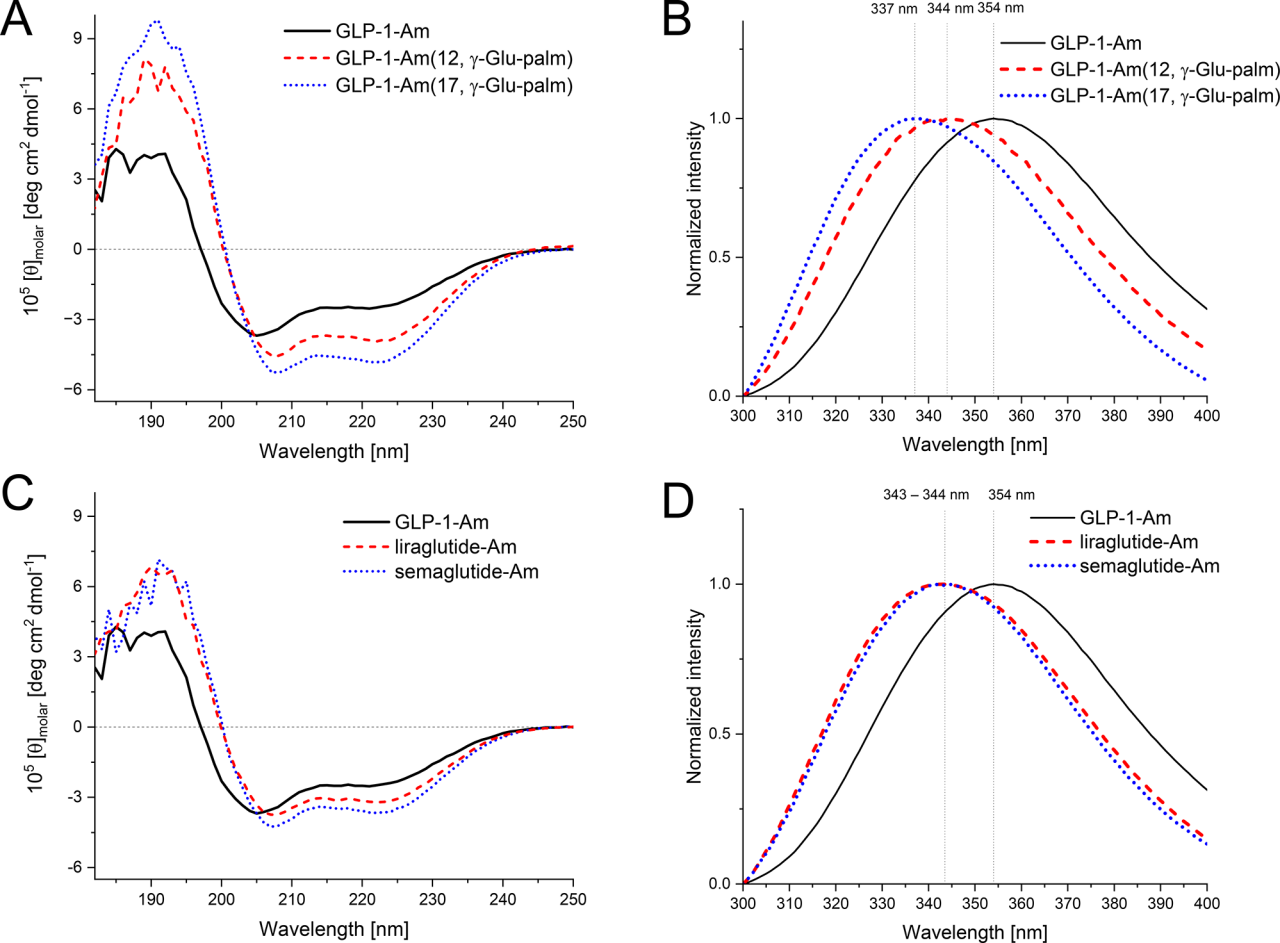
Far-UV CD and intrinsic tryptophan fluorescence emission spectra of freshly dissolved lipidated GLP-1-Am analogues and nonlipidated GLP-1-Am at pH 7.5. All samples were freshly dissolved in 25 mM phosphate buffer at pH 7.5 and measured at 85 μM concentration at room temperature. Far-UV CD spectra (A,C) were converted into molar ellipticity units; fluorescence spectra (B,D) were normalized such that the maximum fluorescence intensity in each spectrum was 1.0.

To investigate the effect of lipidation on the secondary structure of the peptide chain, far-UV circular dichroism (CD) spectra were recorded and analyzed. Far-UV CD spectra of freshly prepared samples at an 85 μM peptide concentration were measured in 25 mM phosphate at pH 7.5 (Figure 2A,C). The algorithm based on the experimentally determined values of mean residue ellipticity at 222 nm (MRE_222_) for fully α-helical and fully coiled protein was used to estimate the α-helix content in the sample (Table 1).^45−47^ As shown in Table 1, the content of the α-helical structure increases by approximately 10–20% upon lipidation of GLP-1-Am. The increase in α-helix content was observed for all lipidated analogues compared to nonlipidated GLP-1-Am; however, its extent was dependent on the position of lipidation and the lipid moiety.

**Table 1:** Estimation of the α-Helical Content of Freshly Dissolved 85 μM GLP-1-Am and Its Lipidated Analogues at pH 7.5

α-helical content estimation based on the mean residue ellipticity value at 222 nm (MRE_222_)	
analogue	α-helix content [%]
GLP-1-Am	21
GLP-1-Am (12, γ-Glu-palm)	34
GLP-1-Am (17, γ-Glu-palm)	42
liraglutide-Am [GLP-1-Am (20, γ-Glu-palm)]	28
semaglutide-Am [GLP-1-Am (20, PEG2-PEG2-γ-Glu-stear)]	32

To further assess the structural differences of lipidated analogues, intrinsic fluorescence spectra reflecting the local environment of the Trp25 residue (with a little fluorescence contribution from Tyr13) were measured (Figure 2B,C). The shift in the fluorescence maximum toward lower wavelengths, which was observed for all lipidated analogues in comparison with nonlipidated GLP-1-Am, indicates a more hydrophobic environment around the Trp25 residue upon lipidation. The prominent reason for this observation is likely the formation of larger and more stable oligomers for the lipidated analogues compared with the nonlipidated GLP-1-Am, which is discussed in the following section.

**Figure 3:**
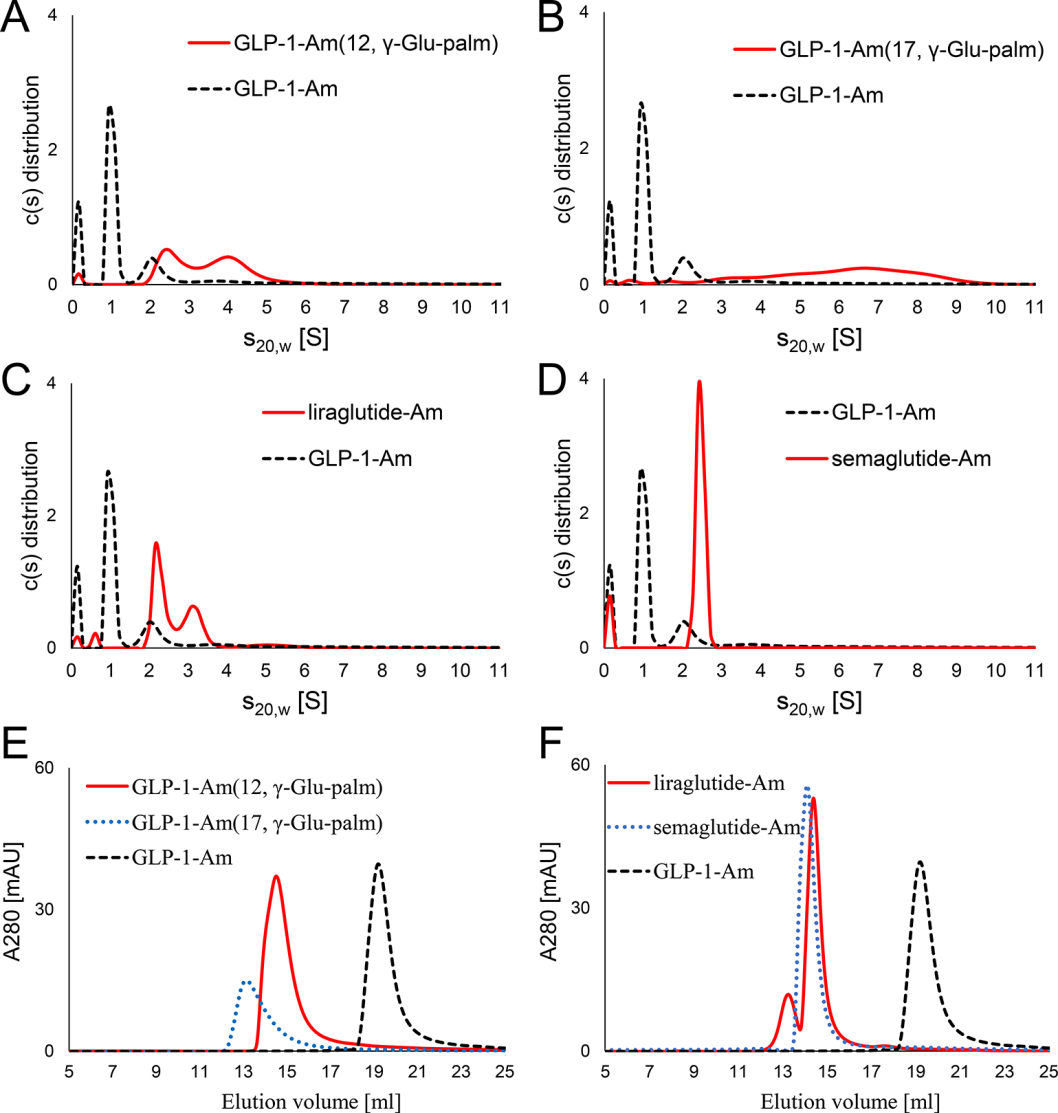
Oligomeric distribution in freshly dissolved samples of nonlipidated GLP-1-Am and lipidated GLP-1-Am analogues at pH 7.5. The oligomeric distributions and populations were investigated using sedimentation velocity—AUC experiments (A–D) and SEC (E,F). Samples were freshly prepared in 25 mM phosphate at pH 7.5 at 85 and 135 μM peptide concentrations for AUC and SEC, respectively. SEC was performed with a Superdex 200 Increase 10/300 column.

Freshly dissolved samples of GLP-1-Am and its lipidated analogues in 25 mM phosphate at pH 7.5 were also analyzed by analytical ultracentrifugation (AUC)—sedimentation velocity experiments, and size-exclusion chromatography (SEC) to establish their oligomeric states, both techniques separating species based on their size and hydrodynamic radii. Figure 3 shows the distributions of oligomeric species of lipidated analogues and nonlipidated GLP-1-Am in solution as they were detected by both techniques. In the AUC experiments, the peak close to 0 S is likely be the monomeric peptide, which is too small to sediment. Nonlipidated GLP-1-Am coexists in mostly a dimeric or monomeric form, with a small percentage of larger (approx. hexameric) oligomers also being observed—Table 2. On the other hand, lipidated analogues were shown to form larger oligomers, with different size distributions of the oligomeric population depending on the position of the lipidation and the nature of the lipid moiety.

**Table 2:** Summary of the Distribution and Population of Oligomers in Freshly Prepared Samples of Nonlipidated GLP-1-Am and Its Lipidated Analogues^a^

	size-exclusion chromatography		sedimentation velocity—analytical ultracentrifugation	
analogue	peak elution volume (s) [mL]	*n*-mer	*s*_20,w_ [S]	*n*-mer
GLP-1-Am*	19.2	1–2	1.00, 2.11	2 (62%), 6 (19%)
GLP-1-Am (12, γ-Glu-palm)	14.5	13	2.64, 4.16	9 (47%), 18 (53%)
GLP-1-Am (17, γ-Glu-palm)	13.2	24	0.74, 1.73, 6.16	1, 5, 32
liraglutide-Am [GLP-1-Am (20, γ-Glu-palm)]	13.2, 14.4	24, 14	0.61, 2.34, 3.19, 5.15	1, 8 (61%), 13 (33%), 28 (5%)
semaglutide-Am [GLP-1-Am (20, PEG2-PEG2-γ-Glu-stear)]	14.1	15	2.42	6–7

a The size of oligomeric species in the samples was estimated from SEC and sedimentation velocity experiments. SEC parameters were calculated using the equations obtained from the Superdex 200 Increase 10/300 column calibration (Figure S9). Sedimentation velocity parameters were obtained directly from the Sedfit program^52,53^ using a continuous *c*(*s*) distribution model. All frictional coefficients in the sedimentation velocity analysis were between 1.3 and 1.4. Percentages in brackets are estimates of the amount of peptide in a particular peak (determined only for easily distinguishable peaks). All experiments were performed in 25 mM phosphate at pH 7.5. *The molecular weight of monomeric GLP-1-Am is below the resolution range of the Superdex 200 Increase 10/300 size-exclusion column.

For GLP-1-Am (12, γ-Glu-palm), two overlapping peaks corresponding to approximately 9-mer and 18-mer were determined by AUC (Figure 3A). The observed overlap of the peaks indicate that these oligomeric species are in rapid equilibrium.^48^ In SEC, GLP-1-Am (12, γ-Glu-palm) elutes as a single broad peak with a theoretical mass of about 13-mer (Figure 3E). This is likely due to the fact that the sedimentation velocity experiments are better able to reflect oligomeric species in rapid equilibrium^48^ compared to SEC, in which the species elute in a broad peak with a theoretical mass in between the masses of the interconverting species.^49−51^ In the case of GLP-1-Am (17, γ-Glu-palm), the distribution of sedimentation coefficients showed a broad unresolved peak over the range of approximately 1–9.5 S and two small peaks at 0 and 0.7 S (Figure 3B). This distribution indicates the presence of trace amounts of smaller species such as monomers and dimers as well as a broad range of larger oligomers which are likely to be rapidly interconverting. Interestingly, peptide concentration-dependent changes were observed in the population of GLP-1-Am (17, γ-Glu-palm) oligomers with a broader range of larger oligomeric species being populated at higher peptide concentrations—Figures S3–S5 and Table S2. This phenomenon was observed only for GLP-1-Am (17, γ-Glu-palm); the populations of oligomer species formed by other analogues did not show similar behavior, at least over the peptide concentration range studied. Liraglutide-Am, GLP-1-Am (20, γ-Glu-palm, was shown to coexist in mainly two oligomeric states, 8-mer and 13-mer, as determined from the sedimentation plot (Figure 3C and Table 2). These species were resolved as distinct peaks in both SEC (Figure 3F) and the sedimentation velocity experiments, suggesting that their interconversion is slow. The oligomeric distribution of liraglutide-Am shows a pH dependence with the larger oligomer being more favorable at lower pH values, Figure S6. Interestingly, two major oligomers of liraglutide-Am are structurally distinct, as is shown in Figure S7. The smaller oligomer (≈8-mer) has a higher content of α-helical structure compared to the larger oligomer (≈13-mer) which shows a prevalence of β-structure. This α-helix to β-structure transition in the oligomeric species is probably the first step leading to further aggregation of the analogue (Figure S8). A similar structural transition in oligomeric species was previously reported in studies on liraglutide.^17,19^ In contrast to other lipidated analogues studied, semaglutide-Am, GLP-1-Am (20, PEG2-PEG2-γ-Glu-stear), which has a different spacer and lipid moiety attached, was shown to be present in solution mostly in the form of a single stable oligomer (Figure 3D,F). Using sedimentation velocity experiments, the size of this oligomer was estimated to be in the range of the hexamer or heptamer. Table 2 illustrates a discrepancy between the size of oligomeric species observed in AUC and SEC. This discrepancy is likely to originate from the overprediction of size by SEC as this technique is dependent on the calibration which is performed with globular protein standards which differ from the behavior of lipidated peptide samples in many aspects.

The results discussed above indicate that the oligomeric distribution of lipidated GLP-1-Am analogues can be regulated not only by the position of lipidation but also by the nature of the linker and the lipid moiety. The reversible oligomerization of GLP-1 analogues is generally considered to be a desirable process as it is an important contributing factor to prolonged stability and slower degradation of peptide therapeutics *in vivo*.^10^ However, there is likely to be a direct link between the distribution and population of oligomeric species in freshly prepared samples and the long-term physical stability of lipidated analogues (e.g., their propensity to aggregate). Therefore, in the following section, the lipidated GLP-1-Am analogues are assessed in terms of their long-term physical stability and tendency to aggregate.

### Aggregation Studies of Lipidated GLP-1-Am Variants

The long-term physical stability of peptide-based therapeutics is essential for the formulation and storage of the drug. Here, aggregation assays and spectroscopic and imaging techniques were employed to assess and compare the physical stability and aggregation propensity of the different lipidated GLP-1-Am variants.

**Figure 4:**
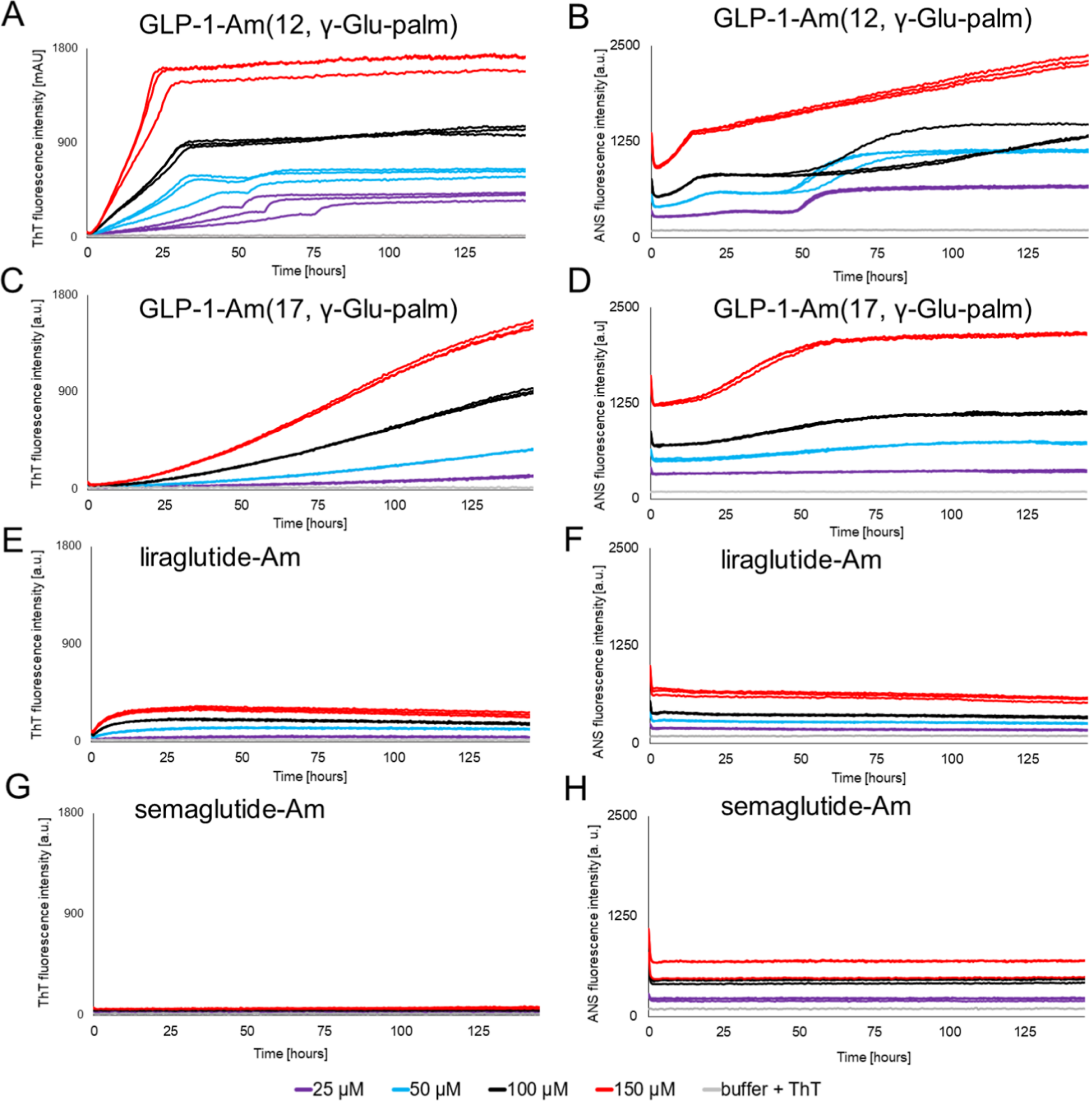
Aggregation of lipidated analogues of GLP-1-Am at pH 7.5—ThT and ANS assays. Different concentrations of GLP-1-Am lipidated analogues were incubated in 25 mM phosphate, pH 7.5, with 50 μM ThT (A,C,E,G) or 250 μM ANS (B,C,D,F,H) at 37 °C with agitation over 145 h. ThT fluorescence was recorded at 482 nm, after excitation at 448 nm, every 30 min. ANS fluorescence was recorded at 482 nm, after an excitation at 355 nm, every 30 min. Each sample in each assay was measured in triplicate in the same plate.

Thioflavin T (ThT) binding assays to detect and follow the formation of amyloid-like fibrils,^54,55^ in combination with assays using 8-anilinonaphthalene-1-sulfonic acid (ANS) to probe the presence of hydrophobic patches in different oligomeric species,^56,57^ were both employed to monitor the aggregation kinetics of lipidated GLP-1-Am analogues. Multiple concentrations of the lipidated GLP-1-Am analogues were monitored over 6 days at pH 7.5 (Figure 4).

The kinetics of amyloid fibril formation *in vitro* from proteins and peptides that are largely monomeric in solution usually follows a nucleation-propagation mechanism, which often results in a typical sigmoidal profile of ThT fluorescence intensity over time.^54^ However, it was previously shown that *in vitro* aggregation of nonlipidated GLP-1-Am deviates from the sigmoidal ThT profile due to the formation of small highly disordered stable oligomers between pH 7 and 8 which competes with the fibrillation process.^58^

None of the lipidated GLP-1-Am analogues studied showed a classical sigmoidal-shaped ThT profile (Figure 4A,C,E,G), and it was, therefore, not possible to fit any of the data to equations describing a nucleation-propagation model.^54^ GLP-1-Am (12, γ-Glu-palm), GLP-1-Am (17, γ-Glu-palm), and liraglutide-Am (GLP-1-Am (20, γ-Glu-palm)) ThT curves all show no or only a very short lag phase. For all the lipidated analogues studied, ANS curves start from nonzero values (Figure 4B,D,F,H), which indicates binding of ANS to oligomeric species and/or to the hydrophobic lipid moiety itself. Additionally, all ANS fluorescence curves show a sharp decrease in intensity during the first hour of incubation. This is caused by an initial temperature equilibration (approximately 22 °C → 37 °C) and the associated change in the viscosity of the sample^59^ since the samples were prepared at room temperature and then transferred to the plate reader thermostated at 37 °C.

The comparison of ThT and ANS profiles for each individual lipidated analogue reveals insights into the number and nature of the steps involved in the transformation of the initial oligomeric species into amorphous or structured aggregates. The aggregation kinetics of GLP-1-Am (12, γ-Glu-palm) show two distinct phases in which the ThT fluorescence intensity increases (Figure 4A). At lower peptide concentrations, the two phases are very distinct, and the gradient of the first phase, between 0 and 50 h, increases with peptide concentration. In addition, the transition between the first and second phase shifts in time with the peptide concentration, with lower concentrations starting the second aggregation phase at later time points. Therefore, the start of the second phase may occur as the concentration of some species formed as part of the first phase accumulates and reaches a critical concentration. Additionally, the first phase contributes to the vast majority of the total ThT fluorescence increase, suggesting that the formation of β-structure occurs in the first phase, while in the second phase, only minor rearrangements of β-structure take place. Consistent with the ThT assays, the aggregation kinetics of GLP-1-Am (12, γ-Glu-palm) followed by ANS show two phases, which are more distinct at lower peptide concentrations. The two-phase aggregation profile observed in ThT and ANS assays was also shown to be pH-dependent—Figure S10, which may indicate a change in the aggregation mechanism or kinetics of its individual steps with pH. The former is supported by the observed differences in morphology of aggregates formed at different pH values (Figure S10).

Samples of GLP-1-Am (17, γ-Glu-palm) show an increase in fluorescence intensity over time in both ThT and ANS assays (Figure 4C,D, respectively).The ThT assay shows a gradual increase in fluorescence intensity without reaching a plateau even at 145 h (Figure 4C), whereas the ANS assay shows an increase in fluorescence during the first 60 h followed by a plateau (Figure 4D). These results suggest that different processes are being probed by ANS and ThT.

Compared to the fluorescence intensity reached in the aggregation assays of GLP-1-Am (12, γ-Glu-palm) and GLP-1-Am (17, γ-Glu-palm), the fluorescence intensities (in both assay types) are significantly lower during liraglutide-Am sample aging, Figure 4E,F. Nevertheless, the ThT curve of liraglutide-Am, Figure 4E, shows a nonzero ThT fluorescence intensity at the start of the assay and a rapid but relatively small increase in fluorescence intensity in the first 10 h for all peptide concentrations tested. In this case, after a maximum ThT intensity has been reached, the fluorescence then decreases slowly. These curves can be explained by the presence of oligomeric species at the earliest time points, which can bind to and increase the fluorescence of ThT. The final slow decay in ThT fluorescence intensity may be caused by photobleaching or by the fact that fibrils formed in the later stages of the assay bind ThT less than oligomeric intermediates which prevail in earlier stages. ANS curves of liraglutide-Am aggregation do not show any significant change in fluorescence intensity in contrast to the ThT curves; this may be explained by a greater sensitivity of ANS to oligomers which are formed rapidly in the solution and therefore causes high fluorescence intensity from the start of the assay.^17,19,56,57^

For semaglutide-Am, ThT and ANS assays did not show any changes in the fluorescence intensity over 6 days of incubation (Figure 4G,H). This observation suggests high physical stability, i.e., no detectable aggregation, of this lipidated analogue over 6 days. The high physical stability is likely to correlate with the formation of a single stable oligomer, which was detected in the freshly prepared solution—Figure 3D,F. It is interesting to compare our results which show no aggregation of semaglutide-Am over 6 days with those on semaglutide (without C-terminal amidation) by Venanzi et al. in which aggregation was observed after several weeks of incubation.^20^ Of note is noteworthy that semaglutide is an equilibrium of monomers and dimers; however, semaglutide-Am studied here adopts a single stable oligomeric species with the size corresponding to a hexamer or heptamer. These results highlight the effect of the C-terminal amidation on GLP-1.

**Figure 5:**
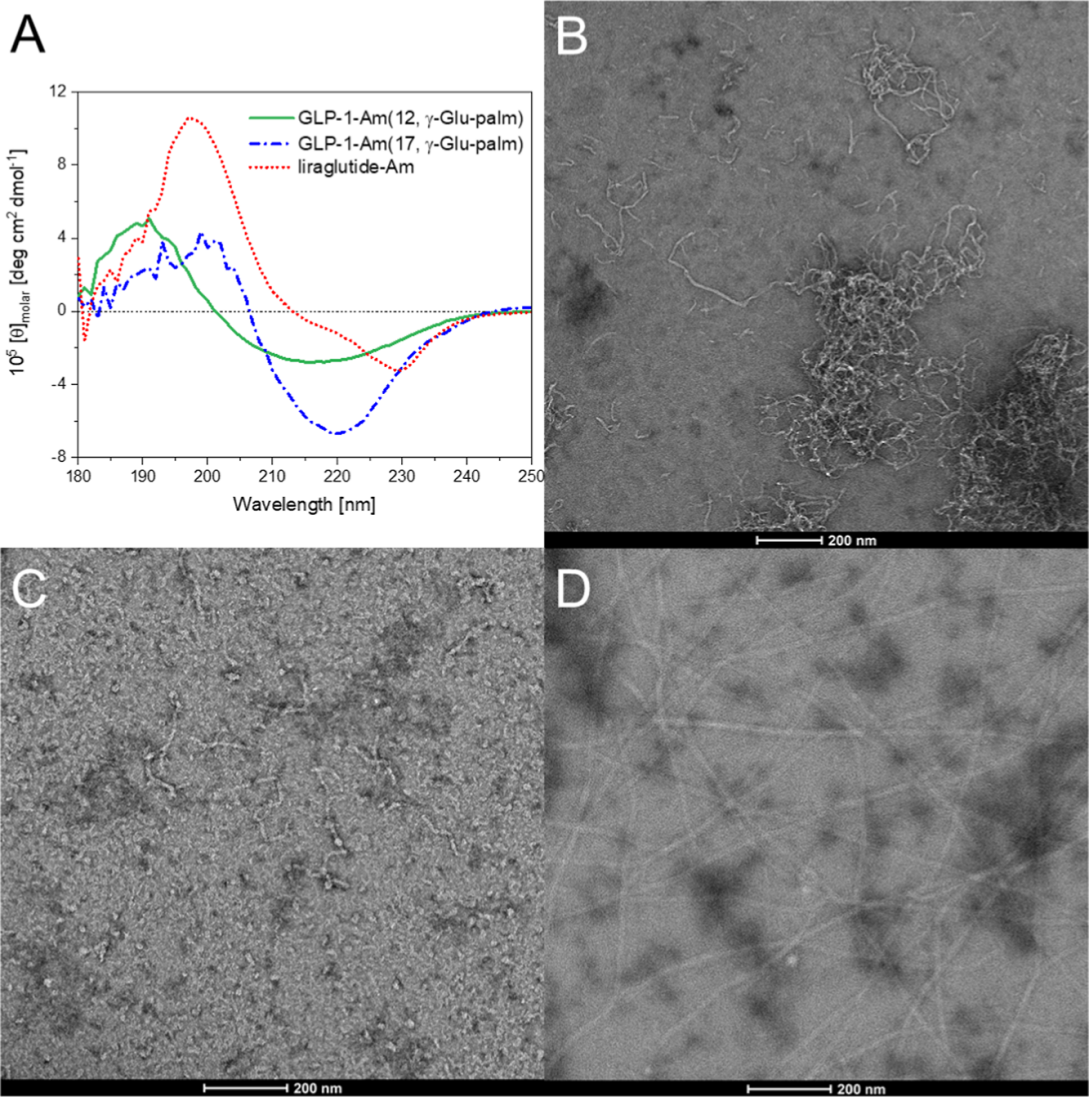
Structure and morphology of aggregates formed at pH 7.5. Far-UV CD spectra of aged samples of GLP-1-Am (12, γ-Glu-palm), GLP-1-Am (17, γ-Glu-palm), and liraglutide-Am (GLP-1-Am (20, γ-Glu-palm)) are depicted in A. For the CD measurements, lipidated analogues were incubated at 85 μM concentration in 25 mM phosphate at pH 7.5 for 8 days with agitation. Samples were measured in a 0.1 cm path length cuvette. The CD signal was converted into concentration-independent molar ellipticity units, [θ]_molar_. TEM images of aged samples of GLP-1-Am (12, γ-Glu-palm) in B, GLP-1-Am (17, γ-Glu-palm) in C, and liraglutide-Am (GLP-1-Am (20, γ-Glu-palm)) in D show the morphology of aggregates formed during the incubation. Samples for TEM imaging were incubated at 25 μM peptide concentration under the same conditions for 8 days prior to application onto the TEM grid.

The morphology and structure of aggregates formed by some of the lipidated GLP-1-Am analogues during aging were investigated using far-UV CD and transmission electron microscopy (TEM) Figure 5. Far-UV CD spectra were measured after 8 days of sample incubation at 37 °C (Figure 5A).For freshly prepared samples, the position of lipidation has only a small effect on the secondary structure of the peptide (Figure 2A,C), whereas the far-UV CD spectra of aged samples are clearly distinct from each other. These observations indicate that the lipidated analogues studied undergo changes in their secondary structure during a long incubation at 37 °C and that the secondary structure of resulting aggregates is affected by the position of lipidation. A BeStSel^60,61^ method was used to estimate the secondary structure content in aggregates of lipidated GLP-1-Am analogues based on their far-UV CD spectra (Table S3). GLP-1-Am (12, γ-Glu-palm), GLP-1-Am (17, γ-Glu-palm), and liraglutide-Am show a decrease in the α-helical structure and an increase in β-sheet content upon aggregation, Tables 1 and S3. Aggregates of GLP-1-Am (12, γ-Glu-palm) and GLP-1-Am (17, γ-Glu-palm) had a higher percentage of disordered regions, whereas for liraglutide-Am, the percentage of disordered regions is low and around 20% of α-helical structure is maintained in the aggregated state. However, care should be taken when interpreting the secondary structure observed in aggregated samples which were analyzed using the BeStSel algorithm as the data sets used in this algorithm do not contain any lipoprotein or lipidated peptide standards which may result in lower accuracy of secondary structure estimation for samples studied in this work.

GLP-1-Am (12, γ-Glu-palm), GLP-1-Am (17, γ-Glu-palm), and liraglutide-Am, which undergo the aggregation detectable in ThT and ANS assays, were imaged using negative-stain TEM (Figure 5B–D). Liraglutide-Am was the only analogue that was observed to form long, rigid, mature fibrils (Figure 5D) in spite of a relatively low fluorescence increase in the ThT assay (Figure 4E). This is caused by a lower binding of ThT dye to the liraglutide-Am fibrils compared to aggregates of other lipidated variants. In contrast, GLP-1-Am (12, γ-Glu-palm) formed thread-like structures; however, these were short, curly, more flexible and tended to assemble into clusters. The existence of short, curly, thread-like aggregates have been previously observed for multiple proteins,^62,63^ and it is likely that these aggregates do not have such a high periodicity of the β-structure as the long, rigid fibrils, but they can be rather a chain of β-structure-rich oligomers. GLP-1-Am (17, γ-Glu-palm) formed short fibril-like fragments as well as small irregular spherical and elliptical oligomers/aggregates (Figure 5C). In addition, these three analogues were monitored by SEC during the aging process with TEM imaging of fractions corresponding to high-molecular weight species (Figure S11). TEM images of isolated high-molecular weight fractions formed after 8 to 72 h showed similar aggregate morphologies to those which were observed after 8 days of aging. Only for liraglutide-Am, the species in high-molecular weight fractions underwent significant elongation during additional aging. Thread-like structures formed by GLP-1-Am (12, γ-Glu-palm) and GLP-1-Am (17, γ-Glu-palm) were not capable of similar elongation observed for liraglutide-Am. The structural and morphological differences between the fibrillar aggregates of GLP-1-Am (17, γ-Glu-palm) and liraglutide-Am were reflected in their infrared and vibrational circular dichroism (VCD) spectra (Figure S12). The IR spectra of both analogues are similar and both show a high content of β-sheet, whereas the signal enhancement in VCD, usually caused by cross-coupling interactions and supramolecular structure periodicity, was observed only from liraglutide-Am fibrils suggesting their higher structural periodicity. This is consistent with the liraglutide-Am fibrils being longer, less curved, and nonbranched.

It was not possible to study the physical stability of the GLP-1-Am (2, γ-Glu-palm) analogue under the same conditions due to solubility issues. GLP-1-Am (2, γ-Glu-palm) is soluble only at around pH 3, and under these conditions, it was observed to rapidly aggregate forming short fibril-like species with high β-sheet content (Figures S13 and S14). This observation highlights the importance of the selection of position of the lipidation site as in the case of lipidation in the N-terminal region of GLP-1, the solubility is significantly decreased, and the rapid formation of β-structure-rich aggregates is greatly promoted. One of the strategies for selecting a suitable lipidation site is lipidation in the proximity of an aggregation-prone region (APR). For GLP-1, the APR is predicted to be mainly between Glu21 and Lys28.^64,65^ In the case of GLP-1 analogues, this strategy seems to be effective as the commercially available analogues liraglutide and semaglutide are both lipidated at Lys20 next to the APR of GLP-1. However, the proximity of APR is likely not the only driving criterion as the lipidation impact on solubility, structure, and bioactivity also play crucial roles.

Overall, the aggregation of lipidated analogues of GLP-1-Am was accompanied by an increase in the β-structure regardless of whether the analogue formed an amorphous aggregate or a fibrillar state (Table 3). The nature and the amount of β-structure formed for each lipidated GLP-1-Am analogue varied, which is likely to be caused by different spatial orientation of β-strands and β-sheets and lower or higher periodicity of structure within the aggregate in each case.^66^ This directly affects the morphology of aggregates formed which ranges from long mature amyloid-like fibrils to short curly species. Therefore, not all GLP-1-Am lipidated analogues form amyloid fibrils but all, except semaglutide-Am, form higher-order structures over time with the final morphologies of the aggregates being greatly variable (Table 3).

**Table 3:** Summarizes the Solubility, Oligomerization, and Aggregation Behavior of all GLP-1 Analogues Studied in This Work, and Figure S15 Presents a Pictorial Representation of the Data for Each Analogue^a^

GLP-1 analogues	pH range of solubility	oligomerization^b^	aggregation
GLP-1-Am	soluble over the entire range studied	dimer and hexamer, less stable, detectable only by AUC, α-helical	amyloid fibrils and disordered oligomers
GLP-1-Am (2, γ-Glu-palm)	soluble only below pH 3	analogue not soluble	analogue not soluble; (at pH 3, there is rapid formation of β-structure-rich fibrils)
GLP-1-Am (12, γ-Glu-palm)	soluble only above pH 7	Two oligomeric species, ca. 9-mer and 18-mer rapidly interconverting, α-helical oligomers	short, curly, flexible thread-like species assembling into clusters
GLP-1-Am (17, γ-Glu-palm)	soluble only above pH 7	wide range of α-helical oligomeric species	short fibril-like fragments and small irregular elliptical oligomers/aggregates
liraglutide-Am [GLP-1-Am (20, γ-Glu-palm)]	soluble only above pH 6	mainly two oligomeric species, ca. 8-mer (α-helical) and 13-mer (β-structure)	long, rigid fibrils
semaglutide-Am [GLP-1-Am (20, PEG2-PEG2-γ-Glu-stear)]	soluble at pH ≤ 3 and above pH 6.4	single, stable, α-helical hexamer or heptamer	none detected

a Table 3 shows the summary of pH-dependent solubility and oligomerization and aggregation behavior at pH 7.5 for lipidated GLP-1 analogues studied. The oligomerization behavior was studied in freshly prepared samples. The aggregation behavior was assessed after 8 days of sample incubation at 37 °C with agitation. Both oligomerization and aggregation behavior were assessed at pH 7.5 unless indicated otherwise.

b Oligomerization state was determined by both AUC and SEC experiments, Figure 3.

Overall, the increased tendency of lipidated analogues to self-assemble into oligomeric species is a desirable phenomenon as it contributes to extended half-life of peptide *in vivo*.^10,67^ However, in general, the subsequent aggregation resulting in an increase in the β-structure, and formation of large aggregates is frequently considered nondesirable. However, there are examples where the slow-release of a peptide-based drug from fibrillar aggregates has been reported and suggested as a therapeutic strategy to obtain controlled release of a drug.^31^ Nevertheless, β-sheet-rich aggregates may cause difficulties with drug distribution due to their size and network-like character or may even trigger an immunogenic response. More investigation in this area is needed.

## Conclusions

This study investigates the effect of lipidation, an established strategy for half-life extension *in vivo* of peptide-based drugs, on the *in vitro* behavior and physical stability of the therapeutic peptide GLP-1. Five lipidated variants differing in both the position and the nature of lipidation were studied and compared to nonlipidated C-terminally amidated GLP-1-Am. Generally, peptide lipidation was found to decrease the solubility of the peptide and limit it to specific pH ranges. Additionally, lipidated analogues were observed to be more α-helical and to form larger and more stable oligomeric species compared to nonlipidated GLP-1-Am in freshly prepared solutions. Interestingly, it was also demonstrated that the size, stability, and distribution of the oligomeric species formed are regulated by both the position and nature of lipidation. However, for the GLP-1-Am (2, γ-Glu-palm) analogue, the lipidation site close to N-terminal region of GLP-1 drastically decreased the peptide solubility, limiting it to pH 3, and resulted in the rapid aggregation into amyloid-like fibrils making this analogue unsuitable for further development.

The aging of lipidated analogues over 6 days was investigated using ThT and ANS assays. Surprisingly, the aggregation kinetics deviated from the sigmoidally shaped ThT profiles typical of nucleation-propagation mechanisms of fibril formation. This is not unexpected given that the lipidated peptides all rapidly form oligomers in solution, which are the starting point of the aggregation reaction, unlike with many amyloid-forming systems where the majority of the peptide is monomeric to begin with. It is also in agreement with the fact that for many of the lipidated GLP-1-Am variants, long-rigid amyloid-like fibrils are not formed but other types of aggregates with less-regular structure are observed. These observations illustrate the great diversity of self-assembly and aggregation processes available to lipidated peptides.

Our findings indicate that the formation of a single stable oligomer in freshly prepared solutions, as is the case for semaglutide-Am (GLP-1-Am (20, PEG2-PEG2-γ-Glu-stear)), results in the greatest physical stability of all the analogues and its lower propensity for aggregation, as opposed to the analogues for which diverse oligomeric states are populated, which aggregate into different species. We believe that our work provides important insights for predicting the stability of lipidated peptide analogues, which, to date, have not been investigated in any depth for many systems. Moreover, it provides findings important for the optimization of GLP-1-based pharmaceuticals, which is highly relevant for future drug development in this area.

## Materials and Methods

### Lipidated Peptides

GLP-1-Am, H-HAEGTFTSDVSSYLEGQAAKEFIAWLVKGRG-NH_2_, molecular weight (MW) of 3355 Da, was purchased from GenScript in the form of an acetate salt with 99.2% purity.

GLP-1-Am (2, γ-Glu-palm), H-HK (γ-Glu-palmitoyl)EGTFTSDVSSYLEGQAAREFIAWRVRGRG-NH_2_, MW: 3878 Da, was purchased from Bachem in the form of an acetate salt with 96% purity.

GLP-1-Am (12, γ-Glu-palm), H-HAEGTFTSDVSK (γ-Glu-palmitoyl)YLEGQAAREFIAWLVRGRG-NH_2_, MW: 3819 Da, was purchased from Bachem in the form of an acetate salt with 95.6% purity.

GLP-1-Am (17, γ-Glu-palm), H-HAEGTFTSDVSSYLEGK (γ-Glu-palmitoyl)AAREFIAWLVRGRG-NH_2_, MW: 3778 Da, was purchased from Bachem in the form of an acetate salt with 96.3% purity.

Liraglutide-Am [GLP-1-Am (20, γ-Glu-palm)], a C-terminally amidated liraglutide analogue: H-HAEGTFTSDVSSYLEGQAAK (γ-Glu-palmitoyl)EFIAWLVRGRG-NH_2_; MW: 3750 Da, was purchased from Peptides International in the form of an acetate salt with >96% purity.

Semaglutide-Am [GLP-1-Am (20, PEG2-PEG2-γ-Glu-stear)], a C-terminally amidated semaglutide analogue, H–H (Aib)EGTFTSDVSSYLEGQAAK (PEG2-PEG2-γ-Glu-stearoyl-COOH)EFIAWLVRGRG-NH_2_; 4113 Da, was supplied by Peptides International in the form of an acetate salt with approximately 96% purity.

All peptides and lipidated analogues were produced using solid-phase peptide synthesis and purified using HPLC. All peptides and lipidated analogues were stored in the form of lyophilized peptide powder at −20 °C.

### Determination of Peptide Solubility

The solubility of nonlipidated GLP-1-Am and its lipidated analogues was tested over a pH range from 2.5 to 8.5. The peptide solubility was tested in the individual buffers differing by 0.5 on the pH scale (i.e., pH 2.5, 3.0, 3.5, 4.0, ...). 500 μL of buffer (25 mM phosphate, citrate, or Tris) of a corresponding pH was added to ca. 0.5 mg of lyophilized peptide powder, gently mixed, and left for ca. 5 min at room temperature before the solution was filtered through a 0.22 μm filter (Millex, PVDF Membrane). Subsequently, the concentration of the peptide was determined spectrophotometrically (Cary 60 UV–vis, Agilent Technologies) using the absorption at 280 nm and a theoretical extinction coefficient of 6990 M^–1^ cm^–1^ at 280 nm.

### Preparation of Fresh Peptide Samples and Sample Aging

Fresh samples were prepared by dissolving the lyophilized peptide powder in a corresponding buffer and subsequent filtration of the sample through 0.22 μm syringe filter (PES membranes, Millex) to remove any nondissolved material or preformed large aggregates originating from the lyophilized powder. The concentration of peptide in the filtered solution was determined spectrophotometrically using the Beer–Lambert law and a theoretical extinction coefficient of 6990 M^–1^ cm^–1^ at 280 nm (ε_280_). During spectrophotometrical concentration determination, an absorption spectrum from 200 to 350 nm was recorded, and the difference of the sample and buffer absorption at 320 nm was checked to determine the contribution of light-scattering to the absorbance, an indicator of aggregate formation. However, no significant light-scattering was observed in any freshly prepared peptide samples, with the exception of rapidly aggregating GLP-1-Am (2, γ-Glu-palm) at pH 3.

Samples for long-term aging experiments were either incubated in a 96-well half-area plate (Corning 3881) or in 1.5 mL plastic microcentrifuge tubes (STARLAB) sealed or wrapped in aluminum foil to protect from sunlight. The incubation was performed at 37 °C with 180 rpm agitation either in a FLUOstar Omega microplate reader (BMG Labtech) or in an Incubator Shaker (Innova 43).

### Circular Dichroism

CD spectra were measured on a Chirascan CD spectrometer (Applied Photophysics). Far-UV CD spectra were measured in a 1 mm path length cuvette, and the measurement was performed with a 1 nm step size and with a 1 nm spectral bandwidth. The resulting spectrum was obtained as an average of three scans, and the spectrum of the pure buffer was subtracted. All measurements were performed at room temperature. The CD machine units (ellipticity-signal expressed in mdeg) were converted to molar ellipticity [θ]_molar_ using the following equation
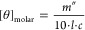
where [θ]_molar_ is the molar ellipticity (with units deg cm^2^ dmol^–1^), *m*^0^ is the CD signal in mdeg (machine units), *l* is the cuvette path length in cm, and *c* is the sample concentration in mol L^–1^.

α-Helical content for soluble (i.e., nonaggregated) samples was estimated using the mean residue ellipticity value at 222 nm (MRE_222_), which was calculated as follows
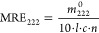
where *m*_222_^0^ is the CD signal in mdeg (machine units) at 222 nm, *l* is the cuvette path length in cm, *c* is the sample concentration in mol L^–1^, and *n* is the number of amino acid residues. α-Helical content was estimated using a method based on a liner interpolation between experimentally determined MRE_222_ values for purely α-helical and purely coiled protein.^45,47,68^ α-helical content is then calculated as
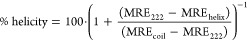
where MRE_222_ is the observed ellipticity at 222 nm, MRE_helix_ is the value for the purely α-helical structure (−35,791 deg cm^2^ dmol^–1^, at 25 °C), and MRE_coil_ is the value for the purely coiled structure (−725 deg cm^2^ dmol^–1^, at 25 °C).

### Intrinsic Tryptophan Fluorescence

Intrinsic tryptophan fluorescence spectra were measured on a Cary Eclipse fluorescence spectrophotometer (Agilent Technologies). Spectra were obtained using an excitation wavelength of 280 nm, and emission spectra were recorded between 300 and 400 nm with a step of 1 nm. Emission and excitation band passes of 10 nm and a voltage on the photomultiplier tube of 550 V were used. Samples were measured in a 120 μL quartz cuvette (Hellma Analytics). Measurements were carried out at room temperature.

### Analytical Centrifugation—Sedimentation Velocity

Sedimentation velocity experiments were performed using a Beckman Optima XL-I Analytical Ultracentrifuge equipped with an An-60 Ti rotor. Samples of 85 μM peptide concentration were freshly prepared before the measurement. After a 2 h temperature equilibration of samples in the centrifuge to 20 °C, the experiment was performed with centrifugation at 50,000 rpm. The interference sedimentation curves were collected as 150 scans (approximately 12 h run time) and fitted to a continuous *c*(*s*) distribution model implemented in the Sedfit program.^48,52,53^ The sedimentation coefficient was corrected for the standard state of the water at 20 °C (*s*_20,w_). The molecular weight and relative amount of each detected species were calculated using the Sedfit program.

### Size-Exclusion Chromatography

Analytical SEC was performed on an KTA FPLC system (GE Healthcare), using a Superdex 200 Increase 10/300 column (GE Healthcare). Samples were loaded using a 200 μL loop. Prior to loading, the samples were filtered through a 0.22 μm filter (Millex, PVDF Membrane) to avoid blocking the column by large aggregates. All samples were eluted at a flow rate of 0.75 mL min^–1^ at room temperature, and UV absorbance detection at 280 nm through a 0.5 cm flow cell was used. A set of globular protein standards (GE Healthcare) was used to construct a calibration curve for the column, Figure S9.

### ThT Binding Assays

Kinetics of aggregation was probed by ThT binding assays using a FLUOstar Omega microplate reader (BMG Labtech). Peptide samples at a given concentration were incubated at 37 °C with 50 μM ThT (Sigma-Aldrich). Peptide samples with ThT were pipetted into a 96-well half a rea plate (Corning 3881) and sealed with tape (Costar Thermowell) to prevent the samples from evaporating. The total volume of sample in a well was 120 μL. Bottom reading of the plate was performed every 30 min with 5 min of shaking prior to each reading (orbital shaker mode at 600 rpm). ThT binding to fibrils and other species was monitored by recording the fluorescence emission at 482 nm after the excitation filter at 448 nm. Fluorescence was measured at a gain of 500 with 8 flashes per well. All samples were measured in triplicate.

### ANS Acid Binding Assay

The exposure of hydrophobic patches in species populated during peptide aggregation was probed using an ANS fluorescent dye (Sigma-Aldrich). Samples were prepared in the wells of a 96-well half a rea plate (Corning 3881) by mixing the peptide samples with ANS to a total volume of 120 μL, in which the final concentration of the ANS dye was 250 μM. To prevent evaporation of the samples, the plate was sealed with tape (Costar Thermowell). The fluorescence measurements were performed using a FLUOstar Omega (BMG Labtech) plate reader, with an excitation filter at 355 nm and an emission filter at 482 nm, at a gain of 500 and 8 flashes per well. The plate was incubated at 37 °C, and readings were taken through the bottom of the wells every 35 min, after 5 min of shaking at 600 rpm, over 6 days. All samples were measured in triplicate.

### Transmission Electron Microscopy

Samples were imaged using a Thermo Scientific Talos F200X G2 Transmission Electron Microscope with an acceleration voltage of 200 kV. 2 μL of the sample was loaded onto a carbon-coated 300 mesh copper grid (EMResolutions or Agar Scientific), which was glow discharged using a Quorum Technologies GloQube system prior to sample application. The sample was dried by blotting, then negatively stained with 2 μL of 2% (w/w) uranyl acetate solution for 15–30 s and dried again.

## Supporting Information Available

Additional characterization of lipidated GLP-1 analogues, isoelectric focusing gel, SEC and spectroscopic characterization of samples, calculation of theoretical net charge, SEC calibration, additional characterization of aggregation and aggregates, graphical representation of the observed behavior, standard p*K*_a_ and p*K*_b_ values used for theoretical net charge calculation, size-characterization of oligomeric species, and prediction of the structure of aggregates (PDF)
