# Supplementary material for: Effect of Lipidation on the Structure, Oligomerization, and Aggregation of Glucagon-like Peptide 1
Source: Bioconjug Chem. 2025 Jan 22;36(3):401–14. doi: 10.1021/acs.bioconjchem.4c00484 (PMC11926786; doi:10.1021/acs.bioconjchem.4c00484)
Supplement: Supplementary file 1 — bc4c00484_si_001.pdf [file bc4c00484_si_001.pdf]

Supporting information

The effect of lipidation on the structure, oligomerization and aggregation of glucagon-like peptide 1

Eva Přáda Brichtová,[†,‡] Irina A. Edu,[†] Xinyang Li,[†] Frederik Becher,[†] Ana L. Gomes Dos Santos[§] & Sophie E. Jackson[†,*]

[†]Yusuf Hamied Department of Chemistry, University of Cambridge, Cambridge, CB2 1EW, United Kingdom

[‡]Now: Institute of Chemical, Environmental and Bioscience Engineering, Technische Universität Wien, Gumpendorferstraße 1A, Vienna, 1060, Austria

[§]Advanced Drug Delivery, Pharmaceutical Sciences, R&D, AstraZeneca, Biomedical Campus, Cambridge, CB2 0AA, United Kingdom

*Correspondence: Sophie E. Jackson. sej13@cam.ac.uk

Determination of isoelectric point of different analogues

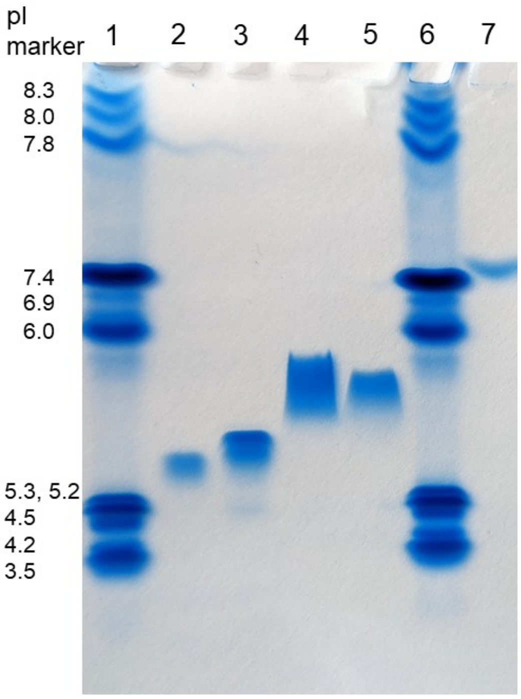

Figure S1: Determination of the isoelectric point values (pI) of lipidated GLP-1-Am analogues. *pI of lipidated analogues was determined using an isoelectric focusing gel pH 3–10 (Invitrogen, Thermo Fisher Scientific). Lanes 1 and 6: pI protein standard markers; lane 2: semaglutide-Am; lane 3: liraglutide-Am; lane 4: GLP-1-Am(17, γ-Glu-palm); lane 5: GLP-1-Am(12, γ-Glu-palm); lane 7: GLP-1-Am (non-lipidated).*

Theoretical calculation of net charge values for different analogues over pH range

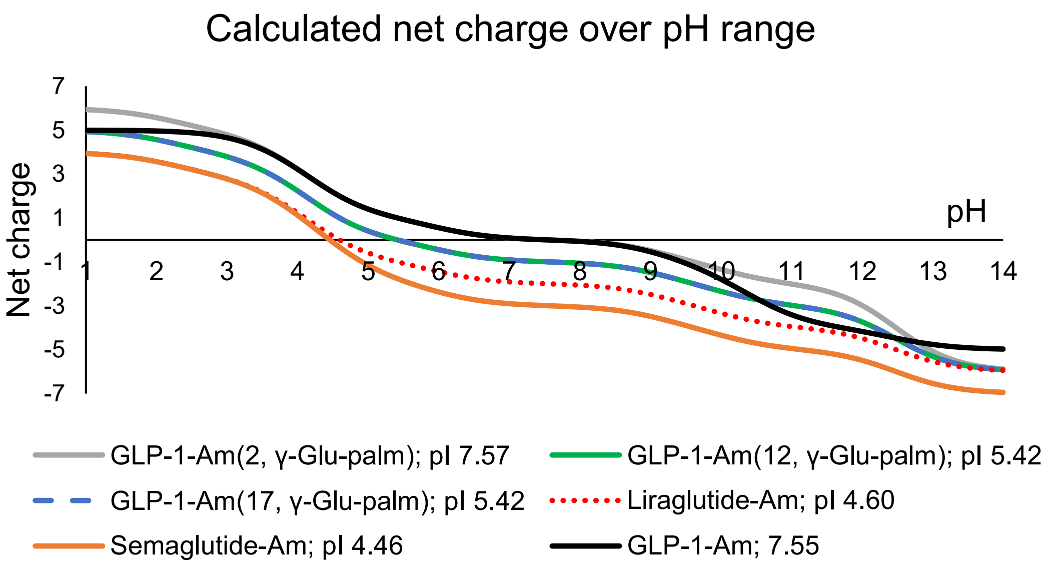

Figure S2: Theoretical net charge values of GLP-1 analogues over a pH range. *The net charge and pI values of GLP-1 analogues were calculated based on* pK_a *and* pK_b *values given in Table S1.*

Acidic or basic group	pK_a	pK_b
Arginine side chain	-	12.48
Lysine side chain	-	10.53
Aspartic acid side chain	3.65	-
Glutamic acid side chain	4.25	-
Glutamic acid α-carboxy group (in linker)	2.19	-
Histidine side chain	-	6
Histidine α-amino group (N-terminus)	-	9.17
Stearic diacid (semaglutide-Am lipid)	4.9	-
Tyrosine side chain	10.07	-

Table S1: Standard pK_a ***and*** pK_b ***values of ionizable groups present in GLP-1 analogues.*** *Standard* pK_a *and* pK_b *values[1] were taken with the approximation assuming that these do not change with the environment of the respective groups in the peptides.*

Theoretical net charge calculation. To plot the net charge vs pH curve of a peptide, the charge on the positively charged amino acid residues was calculated via the following expression, derived from the Henderson–Hasselbalch equation:

$$\sum_{i=1}^{n} \frac{1}{1 + 10^{pH - pK_b}}$$

where the sum is over all the positively charged residues. Similarly, for the charge on the negatively charged amino acid residues the following expression was used:

$$\sum_{i=1}^{n} \frac{-1}{1 + 10^{pK_a - pH}}$$

where the sum is over all the negatively charged residues. The net charge of the peptide at any pH was computed as the sum of the positive and negative charges at that pH and the isoelectric point of the peptide is given by the pH value at which the net charge is zero.

Peptide concentration dependence on GLP-1-Am(17, γ-Glu-palm) oligomer distribution

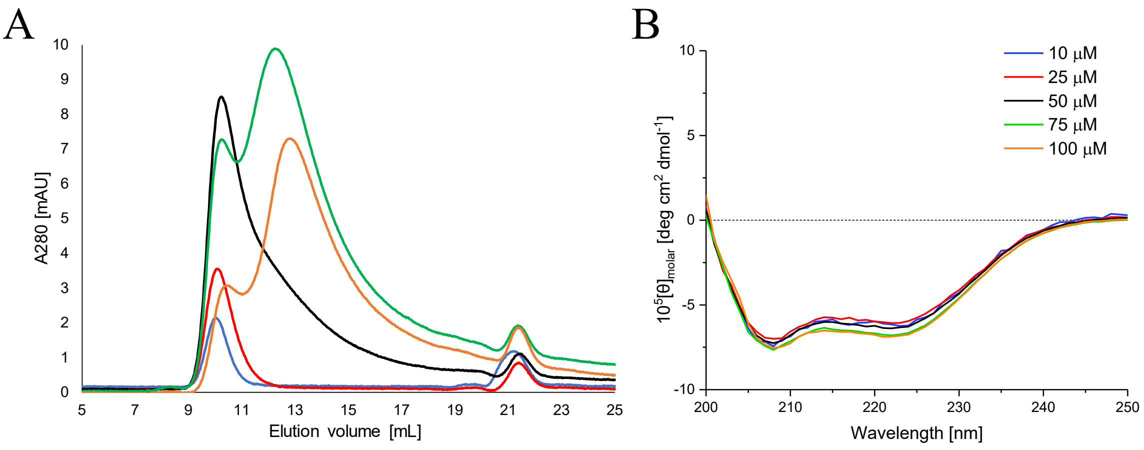

Figure S3: Peptide concentration-dependent distribution of GLP-1-Am(17, γ-Glu-palm) oligomeric species at pH 7.5. *(A) SEC chromatograms of freshly prepared GLP-1-Am(17, γ-Glu-palm) samples at 10, 25, 50, 75, and 100 µM in 25 mM phosphate, pH 7.5. Samples were analysed using a Superdex200 Increase 10/300 column. (B) Far-UV CD spectra of freshly prepared GLP-1-Am(17, γ-Glu-palm) samples at 10, 25, 50, 75, and 100 µM in 25 mM phosphate at pH 7.5. All samples were measured in a 0.1 cm pathlength cuvette. The CD signal was converted into molar ellipticity, $[\theta]_{molar}$, units. The legend located in the top right corner applies to both panels A and B.*

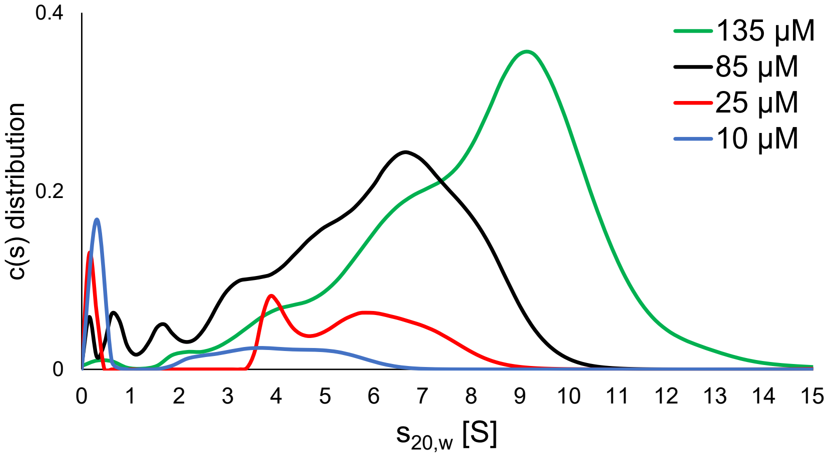

Figure S4: A sedimentation plot of freshly prepared samples of GLP-1-Am(17, γ-Glu-palm) at different peptide concentrations. *Samples of different concentration (from 10 to 135 µM) were prepared in 25 mM phosphate at pH 7.5 prior to the experiment. After 2-hour temperature equilibration to 20 °C, the experiment was performed with centrifugation at 50 000 rpm using Beckman Optima XL-I Analytical Ultracentrifuge. The interference sedimentation curves were collected as 150 scans (approx. 12 h run) and fitted to a continuous c(s) distribution model implemented in a Sedfit program. The sedimentation coefficient was corrected for the standard state of water at 20 °C ($s_{20,w}$).*

Sedimentation velocity: GLP-1-Am(17, γ-Glu-palm) at pH 7.5				
Concentration [μM]	Sedimentation coefficient $s_{20,w}$ [S]	MW estimation [kDa]	Estimated no. of monomers in oligomer	Frictional coefficient
10	4.08	66.7 (60 %)	17–18	1.31
25	4.10<br>6.35	66.8 (26 %)<br>128 (65 %)	17–18<br>34	1.31
85	0.74<br>1.73<br>6.16	4.95<br>17.9<br>120	1<br>4–5<br>32	1.29
135	8.20	181	48	1.27

Table S2: Sedimentation coefficients and calculated molecular weights of oligomeric species of GLP-1-Am(17, γ-Glu-palm) at different peptide concentrations at pH 7.5. *Sedimentation coefficients correspond to the distributions in Figure S4. Molecular weights were calculated using the Sedfit[2,3] program using a continuous c(s) distribution model. The frictional coefficients were optimized to provide the best fit to the model. Percentages in brackets in the MW estimation column give the proportion of a peptide in an oligomeric state (determined only for easily distinguishable peaks).*

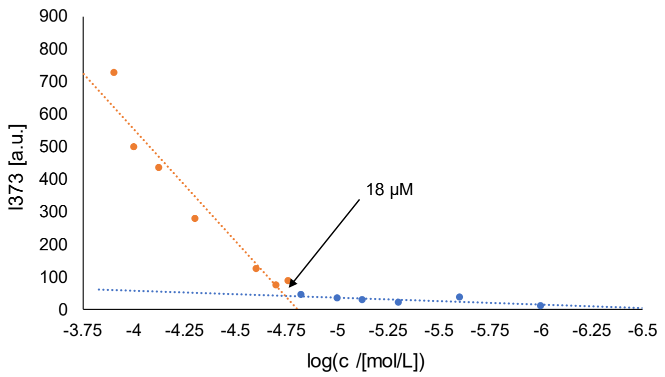

Figure S5: Measurement of the critical aggregation concentration of GLP-1-Am(17, γ-Glu-palm): dependence of pyrene fluorescence on GLP-1-Am(17, γ-Glu-palm) concentration. *Fluorescence intensity at 373 nm was recorded after sample excitation at 339 nm for a range of GLP-1-Am(17, γ-Glu-palm) concentrations (from 1 to 100 μM) with 1 μM pyrene. All samples were freshly prepared in 25 mM phosphate, pH 7.5. Concentrations are plotted as log(c). The intersection of blue and orange line defines the "critical aggregation concentration" which corresponds to approx. 18 μM.*

The sharp increase in pyrene fluorescence above 18 μM GLP-1-Am(17, γ-Glu-palm) concentration is likely to be due to the larger size of the oligomers formed (Figure S4) and therefore greater hydrophobic clusters present in the high concentration oligomers which provide better hydrophobic environment for pyrene. It is also interesting to note that this concentration is close to that observed for the formation of the oligomer detected using SEC at peptide concentrations between 25 and 50 μM (Figure S3A). It should be noted that one must take into the account the additional dilution of the sample during the chromatography, which will result in a decrease from the initial concentration injected onto the column.

The critical micelle/aggregation concentration and/or the presence of micelle-like oligomers in the peptide sample was probed based on changes in pyrene fluorescence, which increases in intensity when the pyrene is in hydrophobic environment, e.g., inside a micelle. Pyrene stock solution (197.6 μM) was added to the peptide sample such that the final concentration of pyrene in the sample was 1 μM. Fluorescence spectra were recorded using a Cary Eclipse Fluorescence Spectrophotometer (Agilent Technologies). Samples were measured in a 120 μL quartz cuvette (Hellma Analytics). The excitation wavelength was 339 nm and the emission spectra were collected between 360 nm and 460 nm in 1 nm steps. The emission and excitation band passes were 5 nm and the voltage on the photomultiplier tube was 600 V. Measurements were carried out at room temperature.

Liraglutide-Am pH-dependence of oligomeric distribution

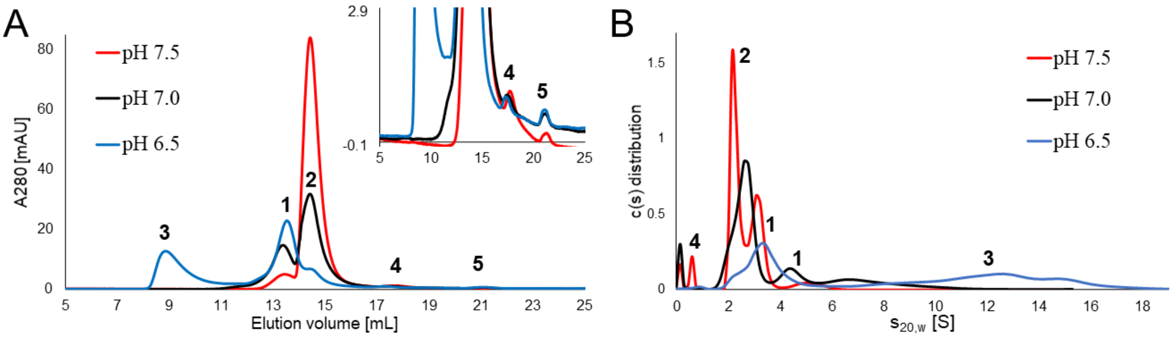

Figure S6: pH-dependent distribution of liraglutide-Am oligomers in freshly prepared samples. *(A) SEC chromatograms of freshly prepared liraglutide-Am samples at 170 µM concentration in 25 mM phosphate buffer of a corresponding pH were measured using a Superdex200 Increase 10/300 column. (B) Sedimentation velocity experiments were performed with freshly prepared liraglutide-Am samples at 85 µM concentration in 25 mM phosphate buffer of a corresponding pH.*

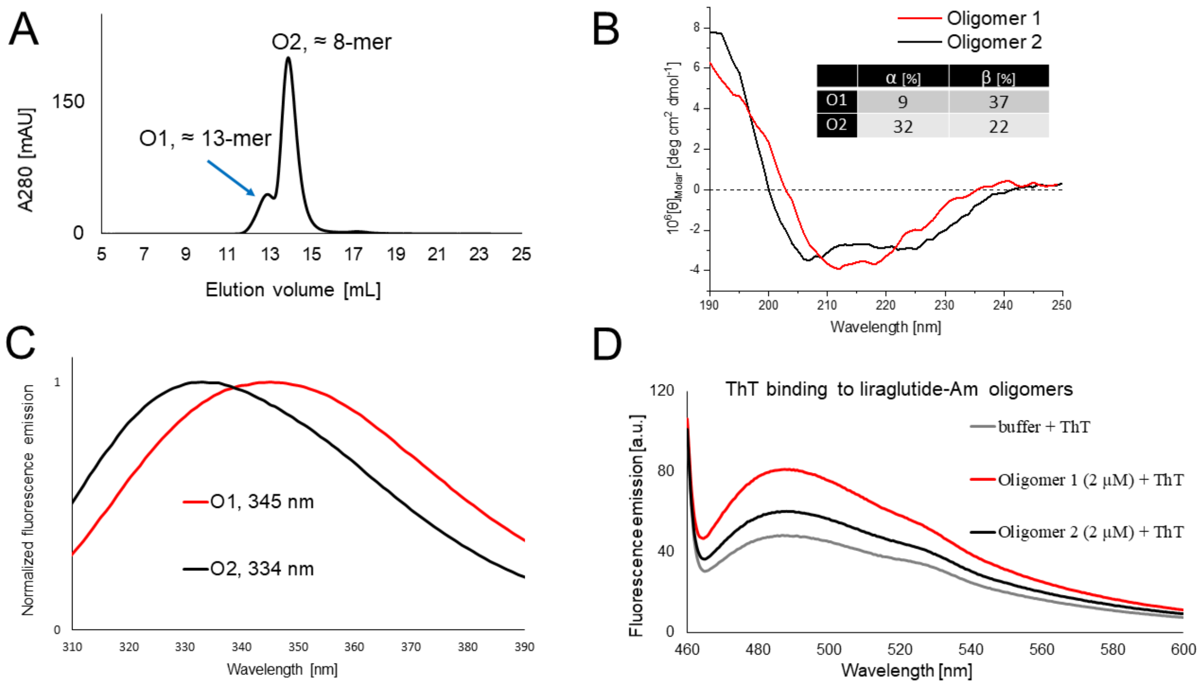

Figure S7: Characterization of liraglutide-Am oligomers. *Liraglutide-Am oligomers 1 and 2 were isolated using SEC (A) and further characterized using far-UV CD (B), intrinsic tryptophan fluorescence (C), and thioflavin T binding experiment (D). Oligomers 1 and 2 were isolated and analysed in 25 mM phosphate at pH 7.5 at 25 °C. The larger liraglutide-Am oligomer O1 was shown to contain more β-structure which correspond to increased thioflavin T fluorescence. Surprisingly, the hydrophobic Trp25 residue is more buried in the smaller oligomer O2 with higher content of α-helix as was shown by intrinsic fluorescence.*

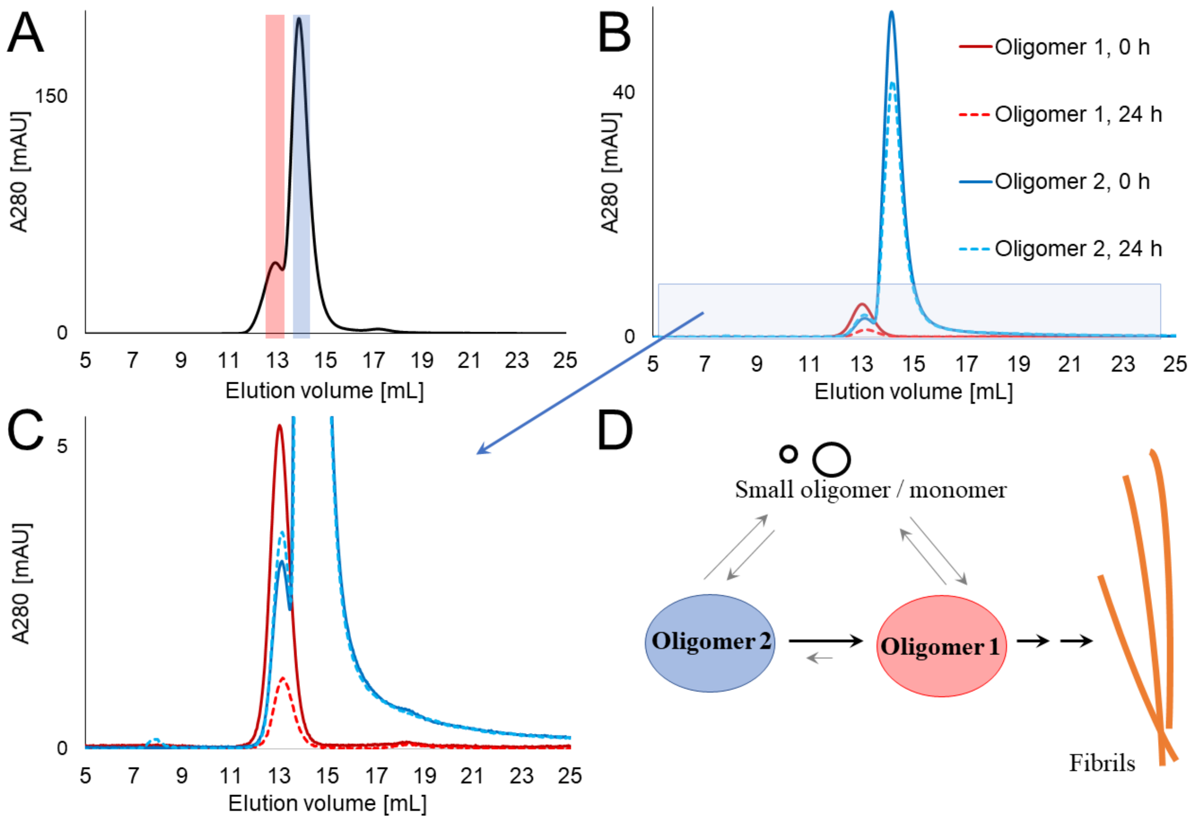

Figure S8: Stability of liraglutide-Am oligomers investigated using size-exclusion chromatography. *Size-exclusion chromatography experiments were performed in 25 mM phosphate at pH 7.5, using a Superdex200 Increase column at room temperature. Samples were filtered through 0.22 µm membrane filter prior to injection onto the column. Initial concentration of liraglutide-Am was 200 µM (chromatogram in A). Isolated fractions (from A) were either immediately reinjected onto the column or left for 24 hours at room temperature before reinjection (B). Panel C shows a magnification of the area in light blue rectangle from (B). A scheme of liraglutide-Am oligomeric species and their mutual conversion is depicted in (D).*

Calibration curve for Superdex 200 Increase 10/300 column

Globular protein standards (GE Healthcare) were used for column calibration, (Fig. S9C). The gel phase distribution parameter, K_{av}, of each protein standard was calculated as

$$K_{\mathrm{av}} = \frac{V_{\mathrm{e}} - V_0}{V_{\mathrm{c}} - V_0}$$

where V_e is an elution volume of a protein standard, V_0 is the void volume of the column (determined by elution volume of Blue Dextran 2000) and V_c is the total volume of the column, 24 mL for all columns used. The logarithm of the molecular weight of each protein standard was plotted against its K_{av}. A linear regression of this plot was used to determine the molecular weight of any eluting species observed in the peptide experiments. For Stoke's radius (R_{St}), a calibration plot of $\sqrt{-\log(K_{\mathrm{av}})}$ against R_{St} and a linear regression was used.

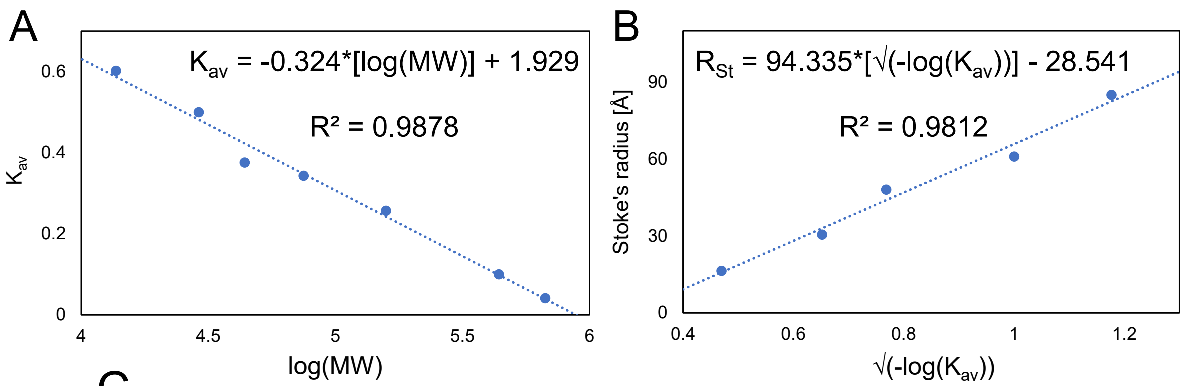

C

Standard	M_W [Da]	Stoke's radius [Å]	$V_{elution}$ [ml]
**Blue Dextran 2000**	N/A (void volume)	N/A	8.1
**Aldolase**	158 000	48.1	12.2
**Ovalbumin**	44 000	30.5	14.1
**Ferritin**	440 000	61	9.7
**Conalbumin**	75 000		13.6
**Thyroglobulin**	669 000	85	8.8
**Ribonuclease A**	13 700	16.4	17.7
**Carbonic Anhydrase**	29 000		16.0

Figure S9: Calibration of a Superdex 200 Increase 10/300 column. *Calibrations on the molecular weight (A) and on the Stoke's radius (B) were performed in 25 mM sodium phosphate buffer at pH 7.5 using a set of protein standards (C) at room temperature. Elution was performed with a flow rate 0.75 mL min*$^{-1}$ *and 100 µL of approximately 100 µM of each protein was injected using a 200 µL injection loop. The blue dotted line is the best fit of the data to a linear regression and the parameters obtained are shown in the equations displayed in the plots (A, B).*

GLP-1-Am(12, γ-Glu-palm) aggregation and aggregate morphology at different pH values

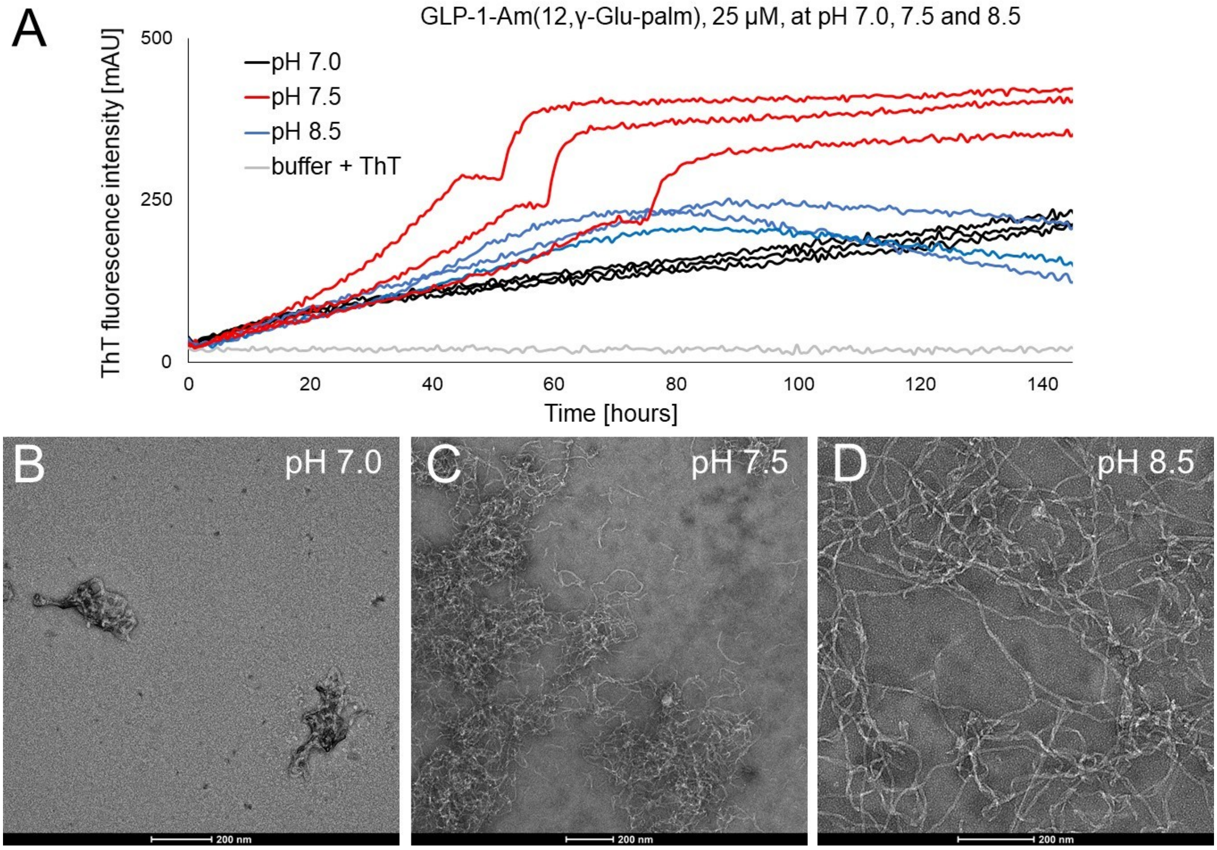

***Figure S10: GLP-1-Am(12, γ-Glu-palm) aggregation kinetics and aggregate morphology at different pH values**. The aggregation of 25 µM GLP-1-Am(12, γ-Glu-palm) in 25 mM phosphate at pH 7.0 and 7.5 and 25 mM Tris at pH 8.5 was monitored by ThT assays (A). Samples with 50 µM ThT dye were incubated at 37 °C with agitation for 145 hours. ThT fluorescence was recorded at 482 nm, after excitation at 448 nm, every 30 minutes. Each sample was measured in triplicate within the same plate. At the end of the assay, the samples were imaged using negative-stain TEM (B, C, D).*

Estimation of secondary structure in aggregated samples of lipidated analogues

Prediction of secondary structure for aged samples					
	α-helix [%]	β-sheet total [%]	antiparallel/parallel β-sheet [%]/[%]	turns [%]	disordered [%]
GLP-1-Am(12, γ-Glu-palm)	10	37	37/0	12	41
GLP-1-Am(17, γ-Glu-palm)	8	43	29/14	10	39
liraglutide-Am	20	45	45/0	19	16

Table S3: Prediction of secondary structure based on far-UV CD spectra of aged samples. *Far-UV CD spectra shown in Figure 5A were analyzed using BeStSel method[4,5] to predict the content of secondary structure. For the CD measurements (A), lipidated analogues were incubated at 85 µM concentration in 25 mM phosphate at pH 7.5 for 8 days with agitation.*

Aggregation of lipidated analogues monitored by size-exclusion chromatography and transmission electron microscopy

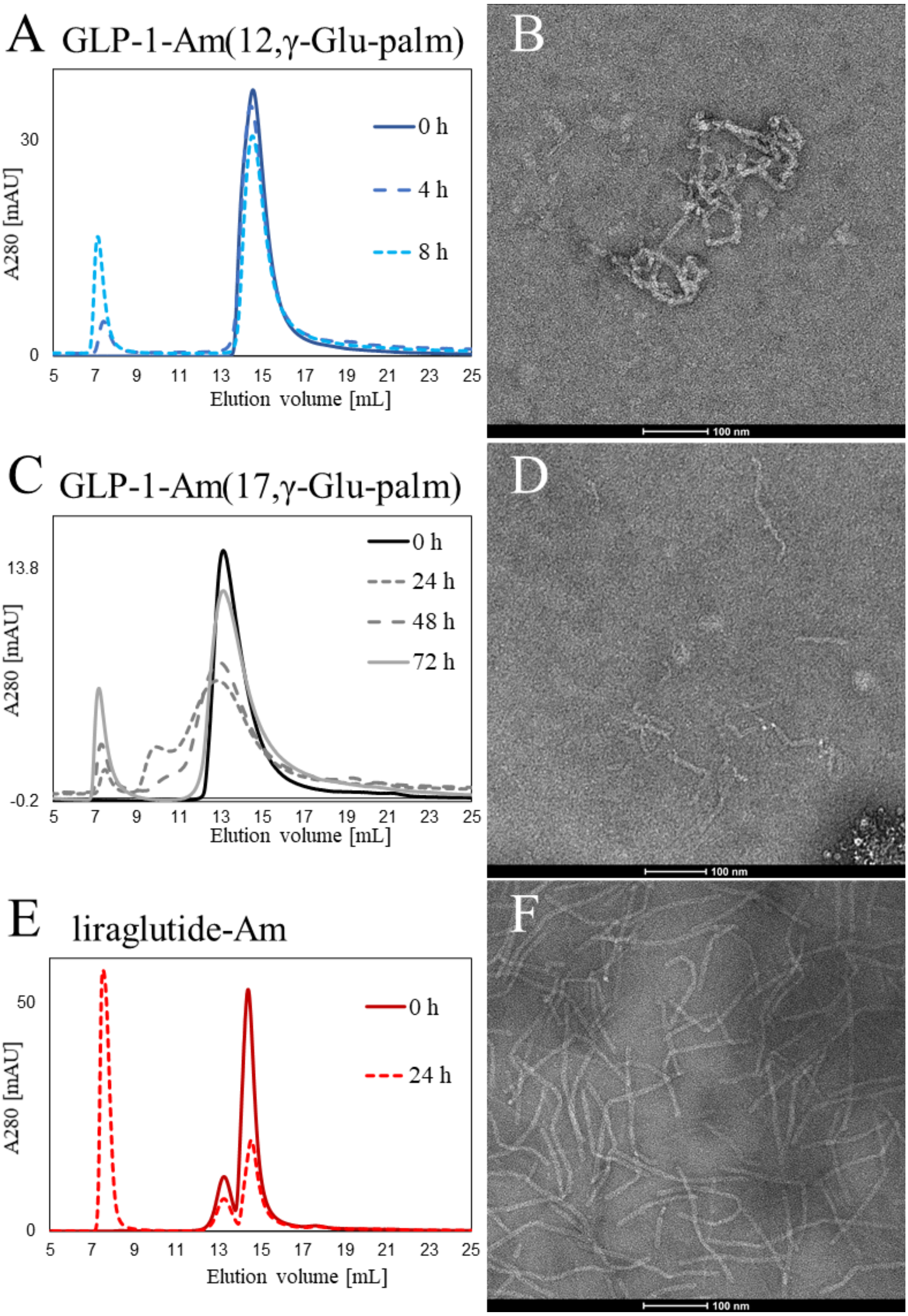

Figure S11: GLP-1-Am(12, γ-Glu-palm), GLP-1-Am(17, γ-Glu-palm) and liraglutide-Am aggregation observed by size-exclusion chromatography and transmission electron microscopy. *Size-exclusion chromatography experiments were performed in 25 mM phosphate, pH 7.5, using a Superdex200 Increase column at room temperature. Both freshly prepared and aged samples (incubated at 37 °C with agitation) were filtered through 0.22 µm membrane filter prior to injection onto the column. For each sample, a fraction in the void volume was isolated, applied onto a carbon-coated copper grid, stained with 2 % uranyl acetate, and imaged using transmission electron microscopy (B, D, F).*

Infrared and vibrational circular dichroism spectra of GLP-1-Am(17, γ-Glu-palm) and liraglutide-Am

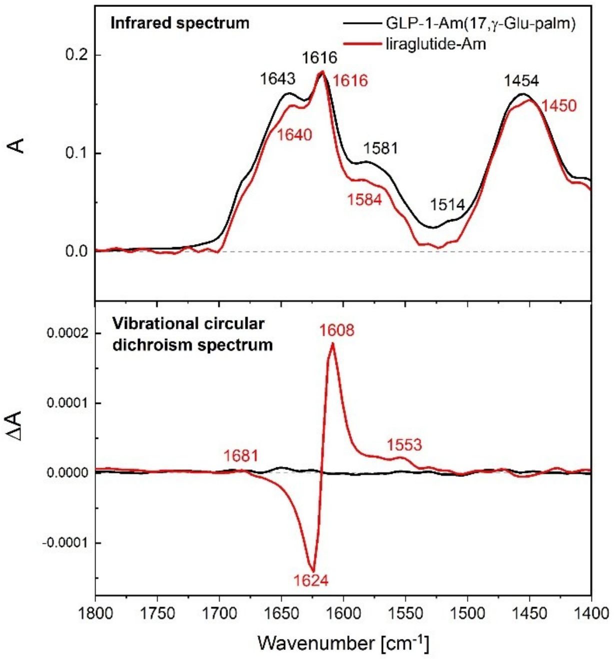

Figure S12: Infrared and vibrational circular dichroism spectra of aged, aggregated samples of GLP-1-Am(17, γ-Glu-palm) and liraglutide-Am*. Spectra were measured in 25 mM deuterated sodium phosphate buffer at pD 7.5 after a 7-day incubation at 37 °C with 180 rpm agitation. The concentration of GLP-1-Am(17, γ-Glu-palm) and liraglutide-Am was 4 mg mL^{-1} (1.06 mM and 1.07 mM, respectively). The infrared absorbance spectra and vibrational circular dichroism spectra have been normalized such that the highest amide I absorption peaks are equal intensity.*

Infrared (IR) and vibrational circular dichroism (VCD) spectra were measured on a ChiralIR instrument (Biotools), using BaF_2 windows, 50 µm optical pathlength, 8 cm^{-1} resolution, and 10 blocks of 2048 scans. Samples of an aggregated GLP-1-Am and its lipidated variants were measured at a concentration of *circa* 4 mg mL^{-1} (*circa* 1.1–1.2 mM) in deuterated 25 mM phosphate buffer at pD 3, 7.5 or 8. All measurements were performed at room temperature. In each case, deuterated phosphate buffer spectra (of corresponding pD) were subtracted as a baseline. Both IR and VCD spectra were normalized to approximately equal the highest amide I absorption.

Aggregation of GLP-1-Am(2, γ-Glu-palm)

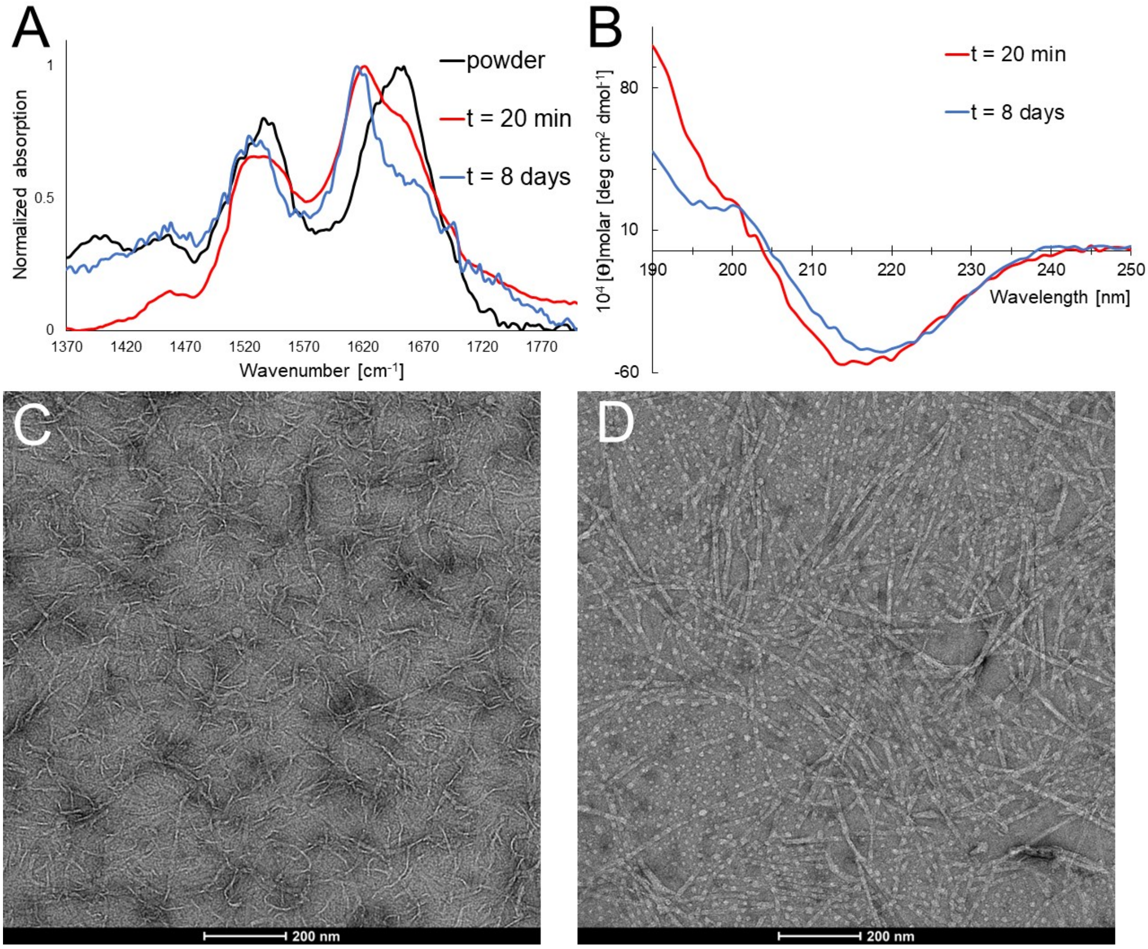

Figure S13: Structure and morphology of species formed by GLP-1-Am(2, γ-Glu-palm). *(A) FT-IR spectra of solid lyophilized samples of GLP-1-Am(2, γ-Glu-palm). Spectra of the original lyophilised GLP-1-Am(2, γ-Glu-palm) as well as samples that were flash-frozen and lyophilized either shortly after dissolution in the buffer or after 8-days of incubation at 37 °C. (B) Far-UV CD spectra of GLP-1-Am(2, γ-Glu-palm) samples at 25 µM in 25 mM phosphate at pH 3. Far-UV CD spectra were measured in a 0.1 cm pathlength cuvette, and the CD signal was converted into concentration-independent molar ellipticity,* $[\theta]_{molar}$*. (C, D) TEM images of uranyl acetate-stained samples. Samples of 25 µM GLP-1-Am(2, γ-Glu-palm) in 25 mM phosphate at pH 3 were applied onto carbon-coated copper grids either circa 20 minutes after sample preparation (C) or after 8-days of incubation at 37 °C with agitation (D).*

Infrared spectra of solid samples were recorded on a FT-IR Perkin-Elmer Spectrum One spectrometer, by loading the lyophilised peptide powder onto the diamond crystal of the apparatus. The spectra were collected using 20 scans, averaged and normalised such that the maximum absorbance represents 100 %. The amide I region (1600–1700 cm^{-1}) was analysed to obtain information about the secondary structure. The aged samples were flash frozen in liquid nitrogen, freeze dried overnight under vacuum in a CoolSafe LaboGene freeze dryer prior to the IR measurements.

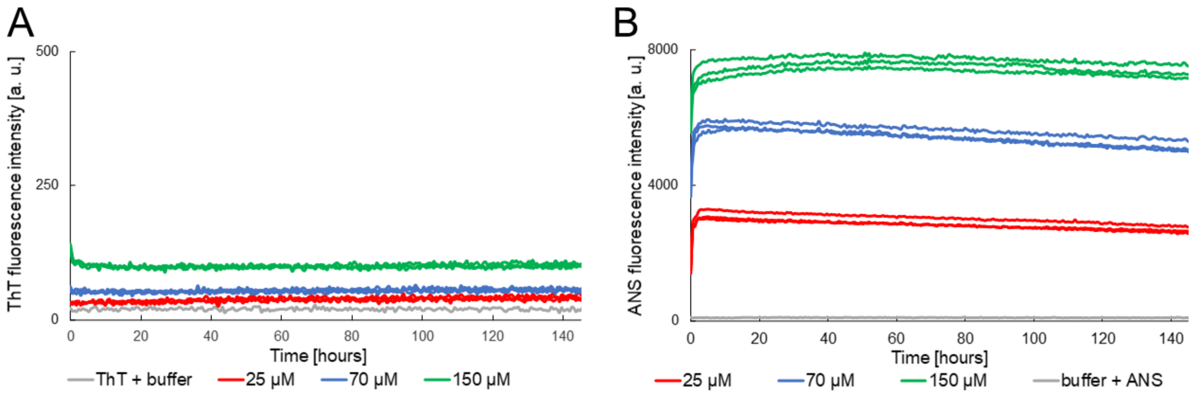

Figure S14: ThT and ANS binding assays of GLP-1-Am(2, γ-Glu-palm) aggregation. *Assays were performed in 25 mM phosphate at pH 3 with either 50 µM ThT (A) or 250 µM ANS dye (B). Three different concentrations of GLP-1-Am(2, γ-Glu-palm) were monitored in both assays – 25, 70 and 150 µM. Samples were incubated for 145 hours at 37 °C with agitation and readings were taken every 30 minutes. Each sample was measured in triplicate in the same plate.*

Graphical summary of oligomerization and aggregation behaviour of lipidated GLP-1 analogues

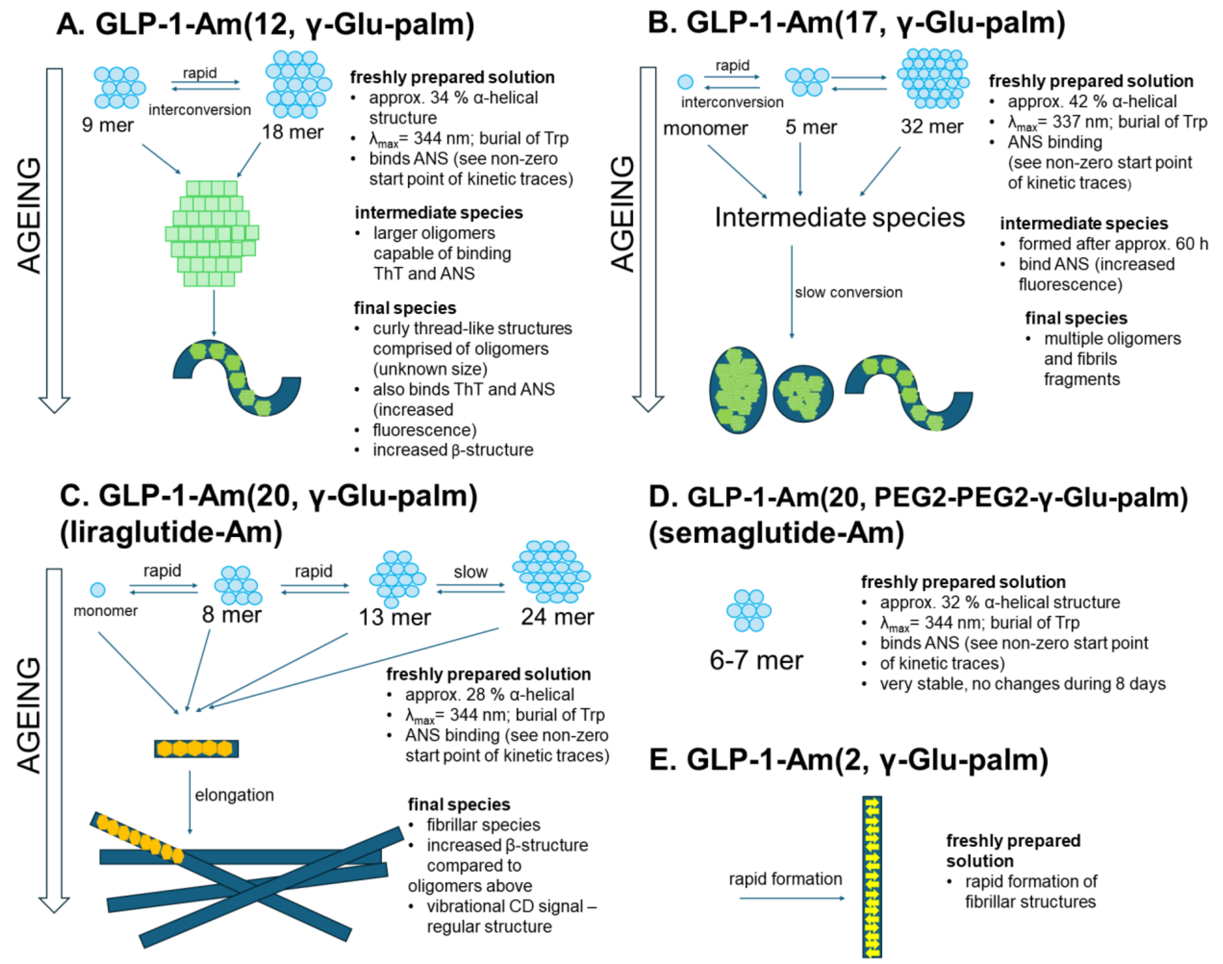

Figure S15: Graphical summary of oligomerization and aggregation behaviour of lipidated GLP-1 analogues. *Analogues A–D were studied at pH 7.5. Behaviour of the analogue in E was described at pH 3 since GLP-1-Am(2, γ-Glu-palm) is insoluble at pH 7.5.*

References:

(1) *CRC Handbook of Chemistry and Physics*; Haynes, W. M., Ed.; CRC Press, 2014. https://doi.org/10.1201/b17118.

(2) Schuck, P. Size-Distribution Analysis of Macromolecules by Sedimentation Velocity Ultracentrifugation and Lamm Equation Modeling. *Biophysical Journal* **2000**, *78* (3), 1606–1619. https://doi.org/10.1016/S0006-3495(00)76713-0.

(3) Lebowitz, J.; Lewis, M. S.; Schuck, P. Modern Analytical Ultracentrifugation in Protein Science: A Tutorial Review. *Protein Science* **2009**, *11* (9), 2067–2079. https://doi.org/10.1110/ps.0207702.

(4) Micsonai, A.; Wien, F.; Bulyáki, É.; Kun, J.; Moussong, É.; Lee, Y.-H.; Goto, Y.; Réfrégiers, M.; Kardos, J. BeStSel: A Web Server for Accurate Protein Secondary Structure Prediction and Fold Recognition from the Circular Dichroism Spectra. *Nucleic Acids Research* **2018**, *46* (W1), W315–W322. https://doi.org/10.1093/nar/gky497.

(5) Micsonai, A.; Moussong, É.; Wien, F.; Boros, E.; Vadászi, H.; Murvai, N.; Lee, Y.-H.; Molnár, T.; Réfrégiers, M.; Goto, Y.; Tantos, Á.; Kardos, J. BeStSel: Webserver for Secondary Structure and Fold Prediction for Protein CD Spectroscopy. *Nucleic Acids Research* **2022**, *50* (W1), W90–W98. https://doi.org/10.1093/nar/gkac345.
